# A land plant‐specific VPS13 mediates polarized vesicle trafficking in germinating pollen

**DOI:** 10.1111/nph.20277

**Published:** 2024-12-01

**Authors:** Surachat Tangpranomkorn, Yuka Kimura, Motoko Igarashi, Fumiko Ishizuna, Yoshinobu Kato, Takamasa Suzuki, Takuya Nagae, Sota Fujii, Seiji Takayama

**Affiliations:** ^1^ Graduate School of Agricultural and Life Sciences University of Tokyo Tokyo 113‐8657 Japan; ^2^ Graduate School of Biological Sciences Nara Institute of Science and Technology Nara 630‐0192 Japan; ^3^ Department of Human Life Science and Design, Faculty of Contemporary Human Life Science Tokyo Kasei Gakuin University 2600 Aihara‐machi, Machida‐shi Tokyo 194‐0292 Japan; ^4^ Japan Science and Technology Agency, Precursory Research for Embryonic Science and Technology Saitama 332‐0012 Japan; ^5^ Graduate School of Bioscience and Biotechnology Chubu University Aichi 487‐8501 Japan; ^6^ Suntory Rising Stars Encouragement Program in Life Sciences (SunRiSE) Kyoto 619‐0284 Japan

**Keywords:** *Arabidopsis thaliana*, cellular polarization, lipid droplet, pollen germination, pollen tube, pollination, vesicle trafficking, VPS13

## Abstract

- Pollen has an extraordinary ability to convert from a dry state to an extremely rapidly growing state. During pollination, pollen receives water and Ca^2+^ from the contacting pistil, which will be a directional cue for pollen tube germination. The subsequent rapid activation of directional vesicular transport must support the pollen tube growth, but the molecular mechanism leading to this process is largely unknown.
- We established a luciferase‐based pollination assay to screen genetic mutants defective in the early stage after pollination. We identified a plant‐specific VPS13, *Arabidopsis thaliana* VPS13a as important for pollen germination, and studied its molecular function.
- *AtVPS13a* mutation severely affected pollen germination and lipid droplet discharge from the rough endoplasmic reticulum. Cellular accumulation patterns of AtVPS13a and a secretory vesicle marker were synchronized at the polarized site, with a slight delay to the local Ca^2+^ elevation. We found a brief Ca^2+^ spike after initiation of pollen hydration, which may be related to the directional cues for pollen tube emergence. Although this Ca^2+^ dynamics after pollination was unaffected by the absence of AtVPS13a, the mutant suffered reduced cell wall deposition during pollen germination.
- AtVPS13a mediates pollen polarization, by regulating proper directional vesicular transport following Ca^2+^ signaling for directional tube outgrowth.

## Introduction

Pollen is usually produced as a quiescent unit but rapidly establishes cell polarity upon pollination to germinate and deliver sperm cells toward an ovule. In plant species with dry stigmas, including the model plant *Arabidopsis thaliana*, pollen coat is mixed with stigma pellicle to form ‘foot’ structure at the pollen‐stigma contact. The structure functions as a conduit for the transfer of necessary liquid for pollen germination (Swanson *et al*., 2004). Pollen hydration reactivates cellular metabolism and induces cell polarity by possible cues such as directional water supply or local Ca^2+^ influx, which ultimately leads to pollen tube outgrowth at the stigma contact site (Lush *et al*., 1998; Wolters‐Arts *et al*., 1998; Iwano *et al*., 2004; Chen *et al*., 2013).

Pollen cells experience drastic physiological changes before germination. Pollen uptake Ca^2+^ after hydration and forms intracellular gradient adjacent to the pollination site, and pollen grains fail to germinate without this local Ca^2+^ elevation (Iwano *et al*., 2004). Asymmetric filamentous actin (F‐actin) network also mediates pollen grain polarity. Common F‐actin pattern found in germinating pollen of many species, including *Arabidopsis*, is the radially aligned F‐actin toward pollen aperture or future germination site (Heslop‐Harrison & Heslop‐Harrison, 1992; Gibbon *et al*., 1999; Zhang *et al*., 2011; Liu *et al*., 2018). More specifically, this collar‐like F‐actin structure is orchestrated by *Arabidopsis* formin homology5 (AtFH5) which relocates onto the plasma membrane at the polarized site and nucleates actin assembly (Cheung *et al*., 2010; Liu *et al*., 2018). The translocation is thought to bring small vesicles to the polarized site (Liu *et al*., 2018). In *Arabidopsis* pollen, secretory vesicles gather at the germination site at *c*. 20 min after pollination (Kandasamy *et al*., 1994). Octameric exocyst complex mediates the exocytosis of vesicles and facilitates the trafficking of cell wall materials for the formation of germination plaque (Hoedemaekers *et al*., 2015; Li *et al*., 2017). Although various cellular events have been reported during pollen polarity establishment, the molecular mechanism leading to such process is still missing.

Several genetic screens involving visible phenotypes such as male sterility (Preuss *et al*., 1993; Hulskamp *et al*., 1995) or distorted segregation (Johnson *et al*., 2004; Boavida *et al*., 2009; Hoedemaekers *et al*., 2015) have been used to isolate mutants with defects in the reproductive process; however, molecular mechanisms specific to the early stages after pollination have rarely been revealed. In this study, we established a forward genetic screening scheme to identify mutants defective in early events after pollination. We found that AtVPS13a mediates pollen polarization by regulating organelle rearrangements and ensuring sufficient vesicle trafficking for pollen germination. AtVPS13a showed polarized localization in germinating pollen grains with exocyst subunits or the secretory vesicle marker. We discuss the possibility that AtVPS13a is the early pollination signal transducer after Ca^2+^ influx, which establishes pollen polarity by guiding vesicle secretion to the pollen germination site.

## Materials and Methods

### Plant growth conditions

Surface‐sterilized *Arabidopsis thaliana* Col‐0 seeds were sown on ½‐strength Murashige & Skoog medium (½MS) containing 0.8% agar, stratified at 4°C in the dark for 3 d and then transferred to a growth chamber set at 22°C with a 14 h : 10 h, light : dark cycle. About 1‐wk‐old seedlings were transferred and grown on soil in a growth room at 22°C with the 14 h : 10 h, light : dark cycle.

### Reporter plants

All oligonucleotides used in this study are listed in Supporting Information Table S1. The promoter region of At4g24570 (Dicarboxylate carrier 2 gene, *c*. 1 kb) was amplified from genomic *A. thaliana* Col‐0 DNA. The purified fragment was cloned into pCambia1300, described in a previous study (Fujii *et al*., 2019), using the In‐Fusion HD Cloning Kit (TaKaRa Bio, Shiga, Japan) after *Hin*dIII/*Bam*HI double digestion of the vector. The *Photinus pyralis* luciferase gene fragment was then cloned into the *Bam*HI/*Sac*I site of the vector to achieve the fusion of the At4g24570 promoter to the luciferase reporter. The construct was introduced into the Col‐0 strain using the *Agrobacterium*‐mediated transformation method (Clough & Bent, 1998). The creation of the pollen calcium reporter line ACT1_pro_:*YC3.6* was reported previously (Iwano *et al*., 2009). The reporter line was crossed with *atvps13a* to generate homozygous *atvps13a* individuals expressing YC3.6 (*atvps13a*/ACT1_pro_:*YC3.6*). To generate dual reporter lines: UBQ10_pro_, mCherry:HSP_ter_, and RabA4B were sequentially cloned into pCAMBIA1300 vector using In‐Fusion cloning technology (TaKaRa Bio) to construct UBQ10_pro_:mCherry:RabA4B:HSP_ter_ cassette. UBQ10_pro_:Venus:RabA4B:HSP_ter_ cassette was cloned into pCAMBIA1300 vector via a similar process. For the R‐GECO1 construct, R‐GECO1:HSP_ter_ was cloned after UBQ10_pro_ in pCAMBIA1300 backbone. *Agrobacterium tumefaciens* containing each plasmid was then used to transform either GT‐VPS13a:Venus plant or SALK_058035, *atvps13a*, plant.

### T‐DNA lines and mutant plants

T‐DNA lines were obtained from the Arabidopsis Biological Resource Center (ABRC). Their stock codes for the lines used in this study are as follows: *cer1*, SALK_008544C; *cer6‐2*, CS6252; *fkp1*, SAIL_675_A04; *atvps13a‐5*, and SALK_058035. For the genetic screen, plants grown from ethyl methanesulfonate (EMS)‐treated seeds were referred to as the M_1_ generation. Selfed seeds from each M_1_ individual were collected separately, and about five M_2_ plants from each M_1_ individual were sown on the soil. Pollen from *c*. 2400 M_2_ individuals was screened for pollen activity using the luciferase‐based pollination assay described below. The *atvps13a‐1–4* mutant alleles were generated in this EMS mutagenesis procedure. The *atvps13a‐4* plants used for further luciferase‐based pollination assays and *in vitro* germination assays were BC_1_F_2_ and BC_1_F_3_ progeny derived from a cross between an EMS M_2_ mutant and Col‐0.

### Luciferase‐based pollination assay

Flower buds of reporter plants were emasculated 1 d before the assay. On the day of the assay, a LUMITRAC™ 200 microplate (Greiner Bio‐One, Kremsmünster, Austria) was prepared by filling each well with 280 μl 1% agar, then a pollen‐free reporter pistil was stood in each well. A small drop of 1 mM d‐luciferin + 0.025% Tween 20 was pipetted onto the reporter stigma and allowed to air dry at room temperature for 1 h. The pistil was then pollinated with the pollen of interest. Bioluminescence was counted for 3 s per well at 3 h after pollination for end‐point measurements, or every 3 min for time course measurements, using a TriStar^2^ LB942 Modular Multimode Microplate Reader (Berthold, Bad Wildbad, Germany). The relative increase in bioluminescence was calculated in relation to the bioluminescence of unpollinated reporter pistils.

### Genome deep sequencing for causal mutation detection

F_1_ plants were obtained from the crosses between the EMS M_2_ mutants and Col‐0. F_2_ plants were screened for their lack of ability to induce the pistil bioluminescence of the reporter plants. Pooled genomic DNA samples from *c*. 20 individual homozygous recessive mutants were extracted using the DNeasy Plant Mini Kit (Qiagen) and concentrated by ethanol precipitation. The sequencing analysis and identification of mutations were performed as described in Suzuki *et al*. (2018).

### Aniline blue staining

Pollinated pistils were fixed in ethanol : acetic acid (3 : 1) overnight. Fixed tissues were incubated in 1 N NaOH at 60°C for 30 min before staining in 2% K_3_PO_4_ + 0.01% aniline blue solution in the dark for at least 3 h at room temperature, or for extended periods at 4°C. The specimens were mounted in 50% glycerol and observed using an epifluorescence microscope with the Zeiss filter set 01 (excitation BP 365/12, Emission LP 397). To stain pollen sample after *in vitro* germination, pollen was pelleted after incubation in liquid pollen germination medium (PGM) and resuspended in 2% K_3_PO_4_ + 0.01% aniline blue solution to stain callose before observation by confocal microscope, LSM880 (Zeiss).

### Phylogenetic analysis

Animal, plant, and yeast proteomes were obtained from Ensembl (ftp://ftp.hgc.jp/pub/mirror/ensembl/current_fasta/), the DOE Joint Genome Institute (https://genome.jgi.doe.gov/portal/pages/dynamicOrganismDownload.jsf?organism=Phytozome), and the Saccaromyces Genome Database (https://www.yeastgenome.org/), respectively (Table S2). HMM profiles of Chorein_N (PF12624), VPS13 (PF16908), SHR‐BD (PF06650), and VPS13_C (PF16909) were obtained from Pfam (https://pfam.xfam.org/). We searched each proteome for each of these domains using hmmscan, which is included in the hmmer package (v.3.2.1; http://hmmer.org/). The threshold for E‐value and domE‐value was 1E‐05. In this study, we defined ‘VPS13’ as the proteins possessing all four domains mentioned above. For the construction of the VPS13 phylogenetic tree, sequences of the four domains were extracted from the VPS13 proteins. For each protein, only the domain with the lowest domE‐value from the hmmscan was used when multiple sites were found. Multiple alignments of each domain were done using the Clustal Omega program v.1.2.4 (Sievers *et al*., 2011). All alignments were combined, and gap regions were removed manually. A Bayesian inference was performed using MrBayes v.3.2.6 (Ronquist *et al*., 2012). The Metropolis‐coupled Markov Chain Monte Carlo processes were run for 500 000 generations, and trees were collected every 100 generations. After discarding trees corresponding to the first 25% (burn‐in), the remaining trees were used to generate the consensus phylogenetic tree. Bayesian posterior probabilities were estimated as the proportion of trees sampled after burn‐in.

### Reciprocal cross experiment

Reciprocal crosses were performed between a wild‐type (WT) Col‐0 plant and a heterozygous SALK_058035 *AtVPS13a*+/− mutant plant carrying the kanamycin resistance gene in the middle of the *AtVPS13a* gene (the *atvps1 3a‐5* allele). Immature flowers of each plant were emasculated before anthesis and pollinated with pollen from the other genotype. The experiment was repeated each day on the same plant pair for *c*. 1 wk. Collected seeds were screened on ½MS supplemented with 50 μg ml^−1^ kanamycin, and the seedlings were phenotyped. A chi‐squared test was used to compare segregation of the *AtVPS13a* alleles with the expected distribution by Mendelian's law (1 : 1 ratio of Km_r_ : Km_s_).

### *In vivo* pollination assay

To determine the time pollen grains needed to fully hydrate and germinate on stigmas, pollination assays were performed as described previously (Iwano *et al*., 2014), with a slight modification. The unpollinated WT pistils were fixed with double‐sided tape to glass slides and kept hydrated in 1% agar. The slides were set on an Axiovert 135 (Zeiss) inverted microscope equipped with a micromanipulator, which was used to place individual pollen grains on different papilla cells (one grain per cell). Photographs were taken every minute for 45 min for the following numbers of individual pollen grains: *n*; WT = 102; *atvps13a* = 107; *atvps13b* = 68 pollination events.

### *In vitro* pollen germination

*In vitro* pollen germination was performed as described previously (Li *et al*., 1999) with slight modifications. The PGM contained 18% sucrose, 1 mM CaCl_2_, 1 mM Ca(NO_3_)_2_, 1 mM MgSO_4_, 0.01% boric acid, 1 mM PIPES pH 7.0, and 1% Nusieve™ GTG™ Agarose (Lonza, Basel, Switzerland) in ultrapure water. Pollen was carefully transferred from 3 to 5 open flowers onto a 1 mm‐thick PGM pad. Pollen of different genotypes were germinated on the same germination pad in each replicate. The germination pad was incubated in a humid chamber at 22°C for 1 d before observation. Replicates with Col‐0, *atvps13a‐4*, and *atvps13a‐5* pollen were performed on three different days. The results for *atvps13b* pollen were from germination on three germination pads in 1 d. ANOVA was used to compare the mean germination rates. For live‐imaging and callose deposition experiments, liquid PGM (agarose omitted) supplemented with 10 μM Epibrassinolide (epiBL, E1641; Sigma) was used to increase pollen germination efficiency (Vogler *et al*., 2014). About 15 flowers were collected into 1.5 ml tube and vigorously vortexed in 500 μl PGM + epiBL for 1 min to separate pollen grain from dehisced anther. The tube was incubated at room temperature for 5 min and centrifuged at 500 ***g***, 2 min to pellet pollen grain. The separated pollen pellet was resuspended in a fresh 100–150 μl PGM + epiBL and transferred onto the glass bottom dish for observation under confocal microscope.

### Confocal imaging and pharmacological treatments

All imaging was performed using the Zeiss LSM 880 confocal laser scanning microscope (Zeiss) controlled by Zen 2.3 SP1 FP1 software (v.14.08.201; Zeiss).

Live‐imaging during *in vitro* pollen germination was acquired from pollen/pollen tube in liquid PGM + epiBL. Wide‐field imaging to capture pollen grains polarization and initial germination was imaged by C‐Apochromat ×40/1.1 W Corr M27 water immersion lens at 1‐min intervals. Pollen tube was imaged by Plan‐Apochromat ×63/1.4 Oil DIC M27 immersion lens at 3‐s intervals. For AtVPS13a:Venus dynamics during pollen germination, Venus was excited by 514 nm argon laser, and emission (em.) wavelength was captured at 517–588 nm. In dual‐labeled lines, fluorescent protein variants were excited simultaneously; Venus was excited by 488 nm laser (em. 517–562 nm), while mCherry and R‐GECO1 were excited by 561 nm laser (em. 602–660 nm).

For imaging of pollen tube after drug treatments, Latrunculin B (LatB; L5288; Sigma) or Brefeldin A (BFA, 11861; Cayman Chemical, Ann Arbor, MI, USA) stock solutions in DMSO were first diluted to 2× working concentration in liquid PGM + epiBL before adding to germinated pollen solution on glass bottom dish at 1 : 1 ratio. Pollen tube was imaged after 10–30 min incubation for LatB and after 30–60 min for BFA. DMSO‐treated pollen tube was used as control.

To observe pollen Ca^2+^ dynamic after *in vivo* pollination, unpollinated pistils were prepared as described above for the *in vivo* pollination assay and observed by LSM 880. YC3.6 FRET was observed using Plan‐Apochromat ×20/0.8 M27 objective lens. The YC3.6 protein was excited with 440 nm laser, and fluorescent emissions from ECFP (CFP) and cpVenus (YFP, from FRET) were collected at 464–499 nm and 526–553 nm, respectively. The fluorescence was collected every 10 s for 30–50 min.

To observe callose deposition on the pollen wall, pollen incubated in liquid PGM + epiBL was directly stained by aniline blue solution. Plan‐Apochromat ×20/0.8 M27 objective lens was used to capture wide‐field 3 × 3 tile scanning excited by 440 nm laser and captured at 445–589 nm. Intensities from three Z‐stacks with 3 μm intervals were projected onto a single 2D image by Zen 2.3 software before scoring callose spot.

### Image processing and quantification

To measure AtVPS13a:Venus dynamic at pollen polarized site, a circular region of interest (ROI) with 5 μm diameter beneath the pollen tube emergence site and ellipsoid ROI covering whole pollen grain were drawn. The relative intensities of the polarized site/whole grain were plotted against time to show AtVPS13a:Venus dynamic at future germination site. For dual‐labeled pollen, AtVPS13a:Venus with mCherry:RabA4b or R‐GECO1, the signal intensity was normalized by the value of the first time point.

For measurement of average distance between AtVPS13a:Venus peak signal from pollen tube tip, kymograph of the growing pollen tube was analyzed by Fiji software (Schindelin *et al*., 2012). A moving average of 3 × 3 pixels was applied to raw time‐lapse images to reduce noise. Kymograph was generated from the intensity profile along the middle of the pollen tube (line width = 10) using Zen v.2.3 software, 150 time‐lapse cycles generated 150 pixel‐height kymograph. Grey‐scale kymograph was imported into Fiji and rectangle ROI was drawn to extract the intensity profile (10 pixels‐height binning). Rectangle ROI was moved through the *Y*‐axis at 5 pixels/step creating a total of 29 overlapping bins for each kymograph; then, a distance between the point of highest Venus intensity and the tip of the pollen tube was scored. An average of 29 bins was used as a ‘Average distance from tip’ of each pollen tube.

For measurement of *in vivo* Ca^2+^ dynamic in YC3.6 reporter lines, moving average of 4 × 4 pixels was applied to raw time‐lapse images to reduce noise. Image calculator processing by Zen 2.3 software was performed with the equation (YFP/CFP) × 4000 to get YFP/CFP ratiometric images. Intensity was scaled to min/max scale of the first image, while maintaining a linear relationship, for the ease of identifying Ca^2+^ spikes. A circular ROI with 5 μm diameter was drawn beneath the pollen tube emergence site, and ROI intensity was normalized by the first time point. The Ca^2+^ spike timing was defined by the time in which the relative YFP : CFP ratio reached the first peak after a rapid drop during the early hydration stage. The Ca^2+^ spike timing was used as a reference point of time to compare the Ca^2+^ dynamic of different pollen grains.

### CRISPR/Cas9 edited plants

CRISPR/Cas9‐mediated genome editing was used to generate the *atvps13b* null mutant and the *AtVPS13aΔC2* line. Single guide RNAs (sgRNAs) were designed using the CRISPR‐P 2.0 web‐based service (Liu *et al*., 2017) (http://crispr.hzau.edu.cn). To create a frameshift in *AtVPS13b*, two sgRNA fragments targeting two genomic regions, 5′‐GTTTGTTGAGAGGTCGGTACAGG‐3′ and 5′‐GTGCTTCAAAACTTTATGATGGG‐3′ (protospacer adjacent motifs, PAMs, are underlined) were cloned into pHEE401E to construct an egg‐cell‐specific expression system (following the procedure in Wang *et al*., 2015). To create the in‐frame deletion in *AtVPS13aΔC2*, two sgRNA fragments targeting two genomic regions, 5′‐GACTATCAACAACGAGGCGTAGG‐3′ and 5′‐GGGTCACTTTCGTTTCCTGTTGG‐3′ (PAMs are underlined), were cloned into pHEE401E. The *Agrobacterium*‐mediated transformation of either *Arabidopsis* Col‐0 was done using the floral dip method (Clough & Bent, 1998). Hygromycin‐resistant transformants were screened for gene editing by Sanger's sequencing.

Gene targeting (GT)‐AtVPS13a:Venus plant was generated by sequential transformation method with some modifications (Miki *et al*., 2018). We generated an in‐house vector by sequential cloning of sgRNA‐expressing fragment from pHEE401E and the donor fragment into pGWB backbone containing BASTA resistance gene. The donor fragment generated by overlap extension PCR consisted of 761 bp of AtVPS13a genomic sequence before stop codon fused to Venus and 629‐bp genomic fragment after stop codon. Finally, PAM sequence in the donor fragment was mutated by site‐directed mutagenesis, to avoid being cleaved by Cas9 (synonymous mutation). *Agrobacterium* was used to introduce T‐DNA into the Cas9‐expressing CS69955 background (obtained from the ABRC). BASTA‐resistant transformants were screened for GT allele by genomic PCR.

### Scanning electron microscopy

Stage 12 stamens were fixed in formalin/acetic acid/alcohol and dehydrated in a series of 50–100% ethanol solutions. The dehydrated stamens were critical point dried and pollen grains were manually exposed from the anthers. The specimens were coated with Platinum‐Palladium before being observed with a scanning electron microscope.

### Transmission electron microscopy

Pollinated pistils left at room temperature for 0, 10, and 20 min were cut above the style and immediately frozen in a high‐pressure freezer (BAL‐TEC, Pfäffikon, Switzerland, HPM010). The samples were fixed in 2% osmium tetroxide in anhydrous acetone and kept at low temperatures in a Cryoporter (CS‐80CP; Scinics, Tokyo, Japan). The temperature was maintained at −80°C for 76 h and then gradually increased at a rate of 2°C h^−1^, with holds when reaching −60°C (4 h), −30°C (3 h), and −20°C (4 h). The temperature was then increased to 4°C at 12°C h^−1^ and held for 2 h before transfer to room temperature. The specimen was washed with acetone and embedded in Spurr resin (Spurr Low Viscosity Embedding Kit; Polysciences, Warrington, PA, USA). Ultra‐thin sections (70–80 nm) were prepared using an ultramicrotome (Leica Ultracut UCT; Leica Microsystems. Wetzlar, Germany). The sections were stained in 4% uranyl acetate and lead citrate, and then observed under a transmission electron microscope at 100 kV (JEM‐1010; JEOL, Tokyo, Japan).

### Organelle fractionation

Every sample handling step was performed on ice. At least 60 open flowers randomly picked from five T_2_ GT‐VPS13a:Venus plants were pooled and homogenized by plastic pestle in the homogenization buffer (0.5 M sucrose, 5 mM EDTA, 0.1% (w/v) bovine serum albumin (BSA), 5 mM 2‐mercaptoethanol, Sigma protease inhibitor cocktail, 50 mM Tris–HCl pH 7.5). The homogenate was filtered through 41 and 11 μm nylon filters and then passed through a 5 μm Omnipore membrane filter (JMWP09025; Merck Millipore, Burlington, MA, USA). The resulting filtrate was subjected to differential centrifugation with increasing speeds; 1000 ***g***, 10 min; 15 000 ***g***, 30 min; 15 000 ***g***, 30 min; and 100 000 ***g***, 90 min. Supernatant and pellet from each centrifugation step were kept on ice until used for SDS‐PAGE and western blot analysis.

For sucrose step gradient fractionation, 6.11 g of homozygous T_3_ GT‐AtVPS13a:Venus open flower was vortexed in 0.5 M sucrose 25 mM Tris–HCl pH 7.5 to isolate mature pollen. The solution was centrifuged at 500 ***g*** 2 min 4°C, pollen pellet was transferred into two 1.5 ml tubes and pelleted by centrifugation again. Pollen pellet in each tube was homogenized by pestle for 1.5 ml tube in 700 μl homogenization buffer; 0.5 M Sucrose, 5 mM EDTA, 0.1% (w/v) BSA, 10 mM DTT, proteinase inhibitor cocktail, 50 mM Tris–HCl pH 7.5. Crude pollen extract was subjected to a differential centrifugation program as stated above and supernatants were pooled before ultracentrifugation step. The microsome pellet was resuspended in 535 μl resuspension buffer (6% w/v sucrose, 1 mM EDTA, 2 mM DTT, and 50 mM Tris–HCl pH 7.5) for use in sucrose step gradient centrifugation. The sucrose step gradient (sucrose in resuspension buffer) was prepared in Ultra‐Clear™ centrifuge tube 11 × 89 mm (Beckman Coulter, Brea, CA, USA) starting from the bottom (%w/w): 40%, 1.5 ml; 32%, 2.2 ml; 25%, 2.2 ml; 18%, 2.2 ml; 12%, 1.5 ml; and 6%, 1.5 ml. The pollen microsome fraction was gently loaded onto 6% sucrose fraction and covered on top by a resuspension buffer without sucrose. The gradient was centrifuged at 100 000 ***g*** in SW 41 Ti swinging‐bucket rotor (Beckman Coulter) for 2 h, and each sucrose fraction and interphase were separately collected for SDS‐PAGE and western blot analysis.

### Calcium ionophore treatment

Arabidopsis mesophyll protoplast was prepared and transfected with a plasmid construct 35S_pro_:mNeongreen:C2_VPS13a_:HSP_ter_ as described previously (Fujii *et al*., 2023). About 2 × 10^5^ protoplast cells were incubated in W5 buffer (2 mM MES‐HCl pH 5.7, 5 mM KCl, 125 mM CaCl_2_, 154 mM NaCl) with 5 μM Ca ionophore A23187 (Sigma). After centrifuged at 100 ***g*** for 2 min at room temperature, the protoplasts were resuspended in homogenization buffer (0.5 M sucrose, 5 mM EDTA, 0.1% (w/v) BSA, 5 mM 2‐mercaptoethanol, Sigma protease inhibitor cocktail, 50 mM Tris–HCl pH 7.5) and homogenized by a plastic pestle. The homogenate was centrifuged at 1000 ***g*** for 10 min, 15 000 ***g*** for 30 min, and 15 000 ***g*** for 30 min. Pellets from each centrifugation step were resuspended in a suspension buffer (0.3 M sucrose, 50 mM Tris–HCl; pH 5.7). The resuspended pellet and supernatant were kept on ice until used for western blot analysis.

### Western blotting

Protein samples were run on SDS‐PAGE and transferred onto PVDF membrane (Immobilon‐P; Millipore). The membrane was blocked by PVDF blocking reagent (Toyobo, Osaka, Japan) and rinsed by TBS‐T buffer before incubated with primary and secondary antibodies, respectively. The primary antibodies used were anti‐UGPase (AS05 086; Agrisera, Vännäs, Sweden), anti‐COXII (AS04 053A; Agrisera), anti‐BiP (AS09 481; Agrisera), anti‐Sec21p (AS08 327; Agrisera), anti‐H^+^‐ATPase (AS07 260; Agrisera), anti‐SEC15A (PHY0818A; PhytoAB, San Jose, CA, USA), anti‐GFP (no.: 598; MBL, Tokyo, Japan), and anti‐mNeonGreen (AS21 4525; Agrisera). The secondary antibody used was anti‐rabbit IgG‐HRP conjugate (Bio‐Rad). ECL Prime western blotting detection reagent (Cytiva, Tokyo, Japan) was used with ImageQuant LAS 4000 (GE Healthcare, Chicago, IL, USA) to detect chemiluminescence. Western blotting of all organelle markers and anti‐GFP was performed on three independent experiments of flower protein fractionation (except anti‐Sec21p, one replicate).

### Nano LC‐MS/MS comparative proteomics

Each of the sucrose fractions from 6/12%, 12%, 12/18%, and 18/25% sucrose fraction and interphases were treated with 2% SDS to extract membrane proteins before sending for comparative proteomic analysis service provided by Kazusa DNA Research Institute (Japan).

### Statistical analyses

Statistical analyses were performed using GraphPad software (Domatics, Boston, MA, USA) and Microsoft (Redmond, WA, USA) Excel. For all box plots, the center line shows the median, box limits indicate the 25^th^ and 75^th^ percentiles, whiskers extend 1.5 times the interquartile range from the 25^th^ and 75^th^ percentiles, and data points are plotted as closed circles. All box plots were generated by BoxPlotR (Spitzer *et al*., 2014).

## Results

### Search for novel pollen factors involved in the compatible pollination process

**Fig. 1:**
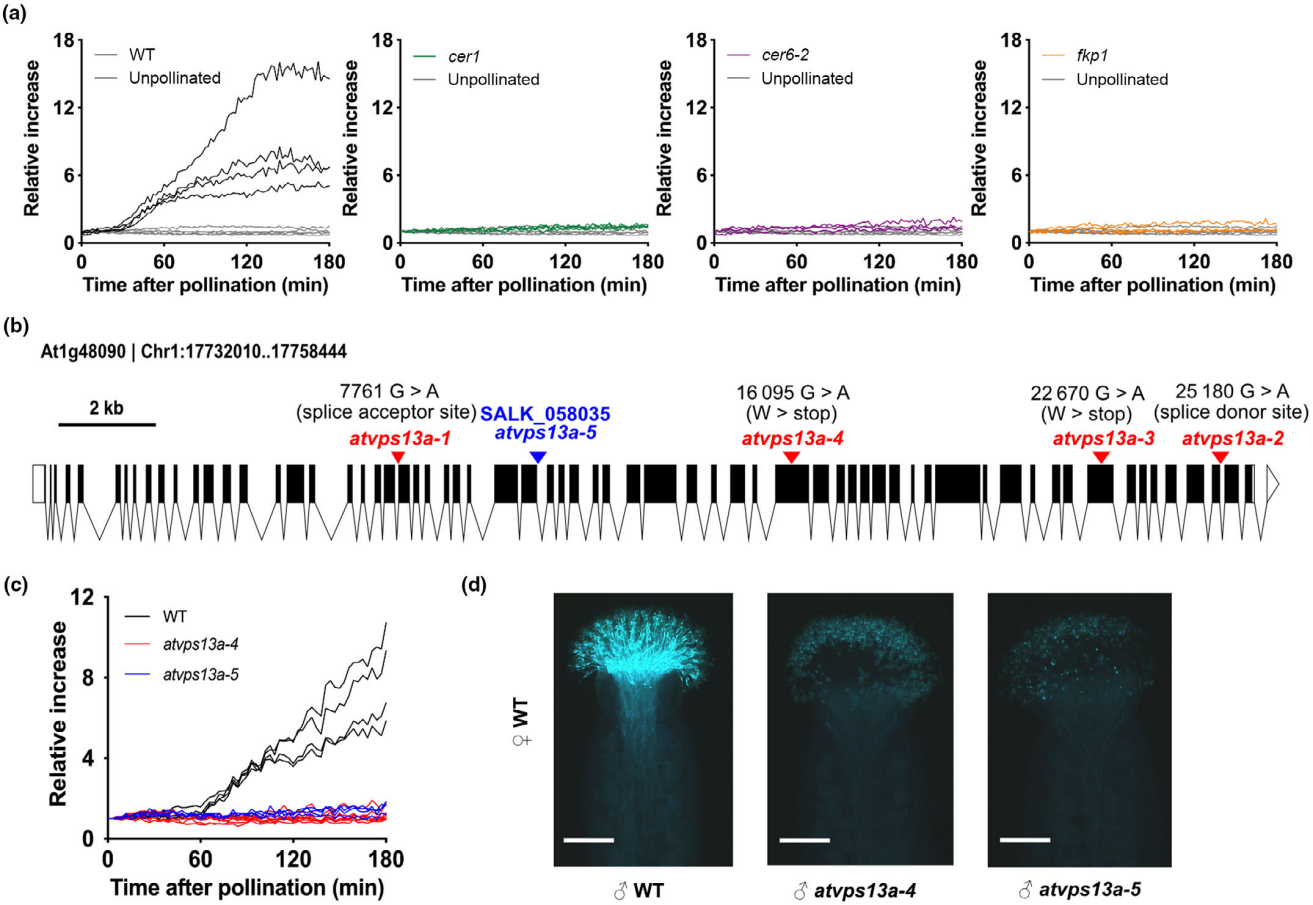
At1g48090 is the causal gene of the noninduction mutant phenotype. (a) Relative increases in the luciferase activity of reporter *Arabidopsis thaliana* pistils after pollinated with wild‐type (WT) and pollen coat mutants (*cer1*, *cer6‐2*, and *fkp1*) pollen. Each line represents one reporter pistil. At least three pistils were pollinated by a pollen donor of each genotype. (b) Gene structure of At1g48090. The exon–intron backbone was generated using http://wormweb.org/exonintron. Arrowheads indicate the locations of ethyl methanesulfonate (EMS)‐generated mutations (red) and the T‐DNA insertion (blue). The *atvps13a‐1* has a nucleotide change at a 3′ splice acceptor site, *atvps13a‐2* has a nucleotide change at a 5′ splice donor site, and *atvps13a‐3* and *atvps13a‐4* contain nonsense mutations. (c) Luciferase‐based pollination assays with WT and *atvps13a* mutant pollen. (d) Aniline blue staining of pistils after pollination with WT or mutant pollen for 3 h. Bars, 200 μm.

Previously, we performed a transcriptomic analysis and identified 398 genes that are induced in stigmas after pollination with compatible pollen (Iwano *et al*., 2014). Among these, the *Dicarboxylate carrier 2* gene (*DIC2*) was upregulated 12.1‐fold postpollination. We then fused its promoter with the *P. pyralis* luciferase gene. This reporter construct was introduced into the WT *A. thaliana* strain Col‐0, creating a system for using reporter pistils in a 96‐well plate assay (Fig. S1; see the Materials and Methods section for details). WT pollen grains induced bioluminescence in this system, whereas pollen grains from known pollen coat mutants *cer1* (Hulskamp *et al*., 1995), *cer6‐2* (Preuss *et al*., 1993), and *fkp1* (Ishiguro *et al*., 2010) did not (Fig. 1a). This indicated that the novel luciferase bioluminescence‐based system can specifically detect pollen‐side mutations involved in the early pollination process.

We screened the pollen of *c*. 2400 M_2_ individuals obtained from EMS mutagenesis and identified 11 independent pollen mutants. Four of these did not induce stigmatic bioluminescence, and seven showed reduced induction compared with WT pollen. We focused our study on the noninduction mutants and used bulked‐segregant analysis with the Mitsucal pipeline (Suzuki *et al*., 2018) to identify the causal mutations (see the Materials and Methods section for details). All noninduction mutants carried either a nonsense mutation or a mutation in a splice acceptor/donor site within the gene At1g48090 (*atvps13a‐1–4*; Figs 1b, S2). Consistent with these EMS‐generated mutants, pollen from a T‐DNA insertion mutant (SALK_058035, *atvps13a‐5*) failed to induce bioluminescence in reporter pistils and did not germinate efficiently on stigmas (Fig. 1c,d). These results indicate that the At1g48090 gene is responsible for the noninduction phenotype. For further experiments, we used *atvps13a‐5*, referred to here as *atvps13a* unless stated otherwise.

### AtVPS13a is a land plant unique form of VPS13

**Fig. 2:**
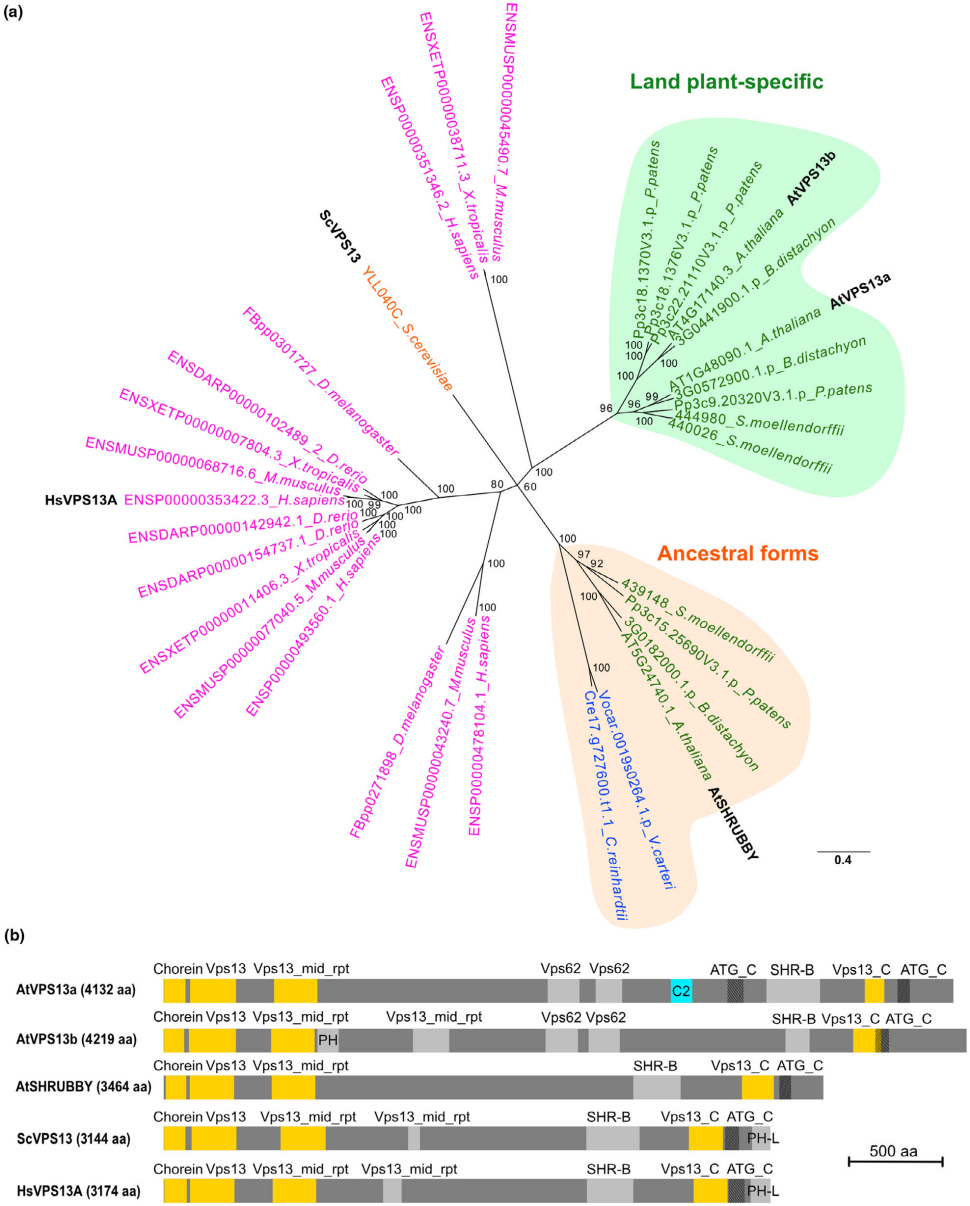
AtVPS13a and AtVPS13b are land plant‐specific VPS13 isoforms with different domains in the middle parts of the proteins. (a) Unrooted phylogenetic tree of VPS13 proteins: magenta, animals; orange, yeast; green, land plants; blue, green algae. Posterior probabilities for Bayesian inference are indicated at each node. The bar indicates substitutions per site. (b) Gene structure of the *Arabidopsis* VPS13s in comparison with yeast ScVPS13 and human HsVPS13A. The gene model was annotated using a hmmsearch of Pfam domains, showing the conservation of VPS13 structural domains at the N‐terminus and C‐terminus (yellow). AtVPS13a carries a unique C2 domain (cyan) not found in other homologs.

The At1g48090 gene was predicted to encode a 4132 amino acid calcium‐dependent lipid‐binding protein with similarity to the yeast Vacuolar Protein‐Sorting associated protein13 (VPS13; GenBank: AJV75928.1). Additionally, we identified two other *A. thaliana* genes encoding VPS13 proteins (At4g17140 and At5g24740). A phylogenetic analysis of VPS13 proteins from various eukaryotic organisms revealed that the At5g24740 protein, named SHRUBBY in a previous study (Koizumi & Gallagher, 2013), may represent the ancestral form (Fig. 2a), due to its molecular size resemblance to yeast and human VPS13A (Fig. 2b). At1g48090 and At4g17140 (designated as *AtVPS13a* and *AtVPS13b*, respectively) belong to a subgroup that specifically diverged in the land plant lineage (Fig. 2a).

All *Arabidopsis* VPS13 proteins retain the VPS13 structural domains at N‐terminus and C‐terminus found in both human HsVPS13A and yeast ScVPS13, indicating that the VPS13 protein structure is broadly conserved (Fig. 2b). VPS13 family members are generally thought to be involved in lipid transport between organelles (Leonzino *et al*., 2021). The hydrophobic cavity structure at the N‐terminus, which is involved in lipid transport function (Kumar *et al*., 2018), was found conserved in the modeled structures of *Arabidopsis* VPS13 proteins (Fig. S3a). An amphipathic helix near the C‐terminus of HsVPS13A, which localizes to lipid droplets (LDs; Kumar *et al*., 2018), also appears to be conserved in *Arabidopsis* VPS13 proteins (Fig. S3b).

Interestingly, only AtVPS13a possesses a putative C2 domain, which is generally involved in Ca^2+^‐dependent protein targeting to biological membranes (Corbalan‐Garcia & Gómez‐Fernández, 2014; Fig. 2b). The predicted protein structures suggest that AtVPS13a may have acquired specific domains essential for supporting land plant‐specific biological events.

### *AtVPS13a* is required for efficient pollen germination

To further specify the role of AtVPS13a in early pollination, we performed reciprocal crosses between heterozygous *AtVPS13a*+/− and WT plants. Our findings indicate that *AtVPS13a* is essential for male gametophytic function but is not required for female function (Fig. S4). We confirmed that *atvps13a* pollen was viable and developed properly into mature tricellular pollen grains, appearing indistinguishable from WT pollen under scanning electron microscopy (Fig. S5).

**Fig. 3:**
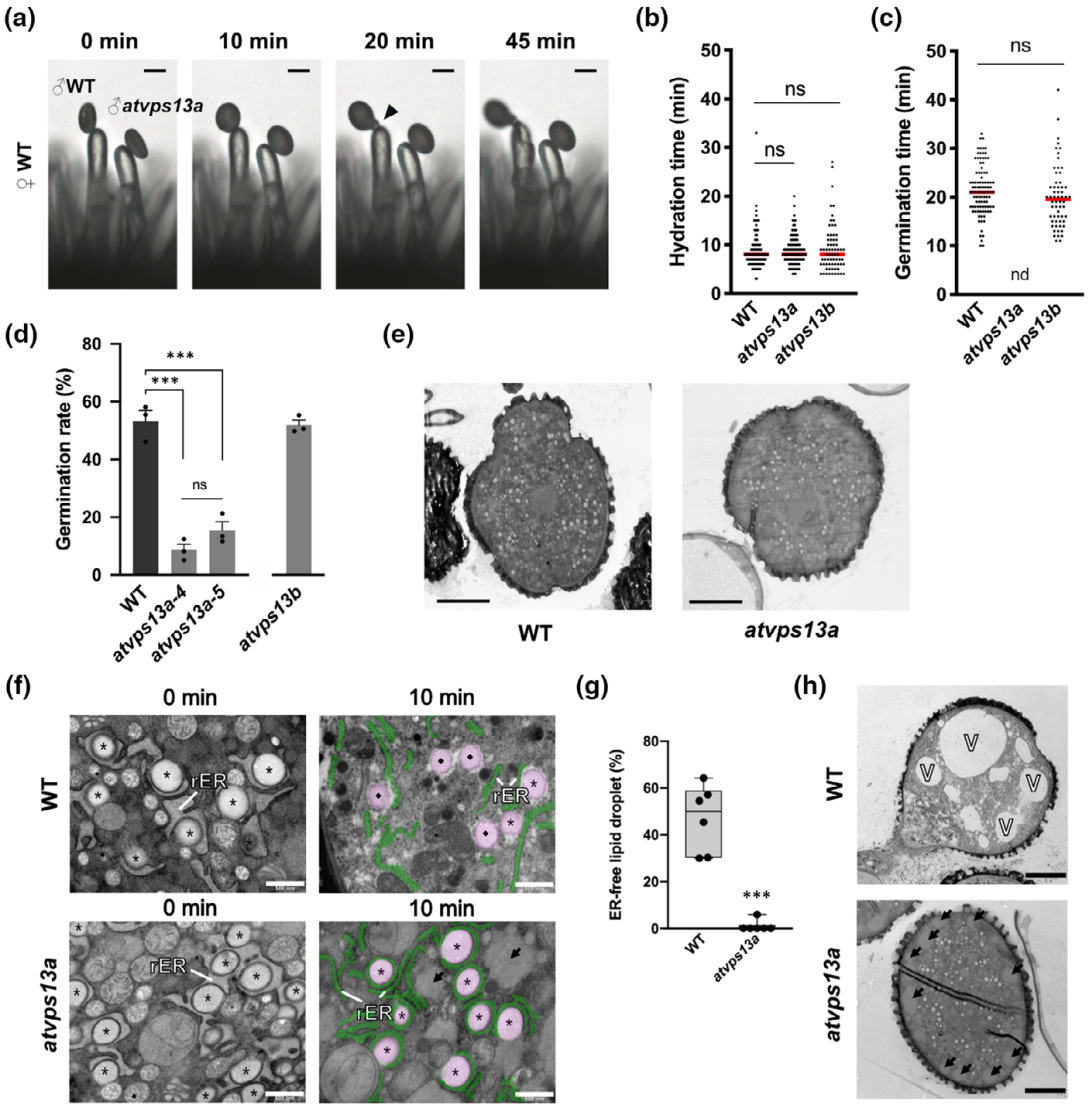
AtVPS13a is the main *Arabidopsis* VPS13 isoform required for lipid droplet‐rough endoplasmic reticulum dynamic during pollen hydration. (a) Representative images from *in vivo* pollination assays, with the arrowhead indicating a germinated pollen tube at 20 min after pollination. (b) Scatter plots of pollen hydration completion times for wild‐type (WT), *atvps13a*, and *atvps13b* pollen on WT stigmas. Red lines, median; ns, not significant (two‐tailed Student's *t*‐test; *P* > 0.05). (c) Scatter plots of pollen germination times for WT, *atvps13a*, and *atvps13b* pollen on WT stigmas. Red lines indicate the median; nd, not detected; ns, not significant (two‐tailed Student's *t*‐test; *P* = 0.136). For (b, c), WT, *n* = 102 pollen; *atvps13a*, *n* = 107 pollen; *atvps13b*, *n* = 68 pollen. (d) *In vitro* pollen germination rates of WT, *atvps13a‐4*, *atvps13a‐5*, and *atvps13b* pollen. Bars indicate the means of replicates, and whiskers indicate SE (*n* = 3663, 3135, 4930, and 3464, respectively, where *n* is the total number of pollen grains from three replicates). Asterisks indicate significant differences by Tukey's multiple comparisons test: *, *P* < 0.05; ***, *P* < 0.001; ns, not significant. (e) Overview of unhydrated pollen at 0 min after pollination by transmission electron microscopy. (f) Transmission electron micrographs of unhydrated pollen at 0 min and hydrated pollen at 10 min after pollination. Pseudo‐colored rER (green) and lipid droplets (LD) (magenta) are labeled in the 10‐min images to visualize LD release. (g) Box plots of the percentages of putative rER‐free lipid droplets in hydrated pollen grains at 10 min after pollination. The center line shows the median, box limits indicate the 25^th^ and 75^th^ percentiles, and whiskers extend 1.5 times the interquartile range from the 25^th^ and 75^th^ percentiles. Asterisks indicate significant differences by two‐tailed Student's *t*‐test: *, *P* < 0.05; ***, *P* < 0.001; *n* = 6 pollen grains. (h) Germinated WT pollen and arrested *atvsp13a* pollen at 20 min after pollination (f, h) rER, rough endoplasmic reticulum; asterisk, rER‐bound lipid droplet; diamond, putative rER‐free lipid droplet; arrow, putative vesicle fusion structure; V, vacuole. Bars: (a) 20 μm; (e, h) 5 μm; (f) 500 nm.

To investigate the defect in *atvps13a* pollen, we used a micromanipulator to transfer pollen grains onto stigmatic papilla cells and observed their hydration and germination processes (Fig. 3a). The time needed for complete hydration of *atvps13a* pollen (mean ± SE, 8.8 ± 0.29 min) was comparable to WT pollen (8.7 ± 0.38 min) (Fig. 3b). However, none of the *atvps13a* pollen grains germinated within the observation period (45 min), whereas 94.1% of WT pollen grains germinated at 21.4 ± 0.52 min (Fig. 3c). Additionally, *atvps13a* pollen grains germinated less efficiently under *in vitro* conditions (Fig. 3d).

No pollen germination defects were observed in a CRISPR/Cas9 genome‐edited *atvps13b* null mutant (Figs 3c,d, S6), suggesting that the closest paralog of AtVPS13a is not required for pollen germination. This is supported by the higher expression levels of *AtVPS13a* compared with *AtVPS13b* or *SHRUBBY* in mature anthers and pollen (Fig. S7).

### The *atvps13a* mutant failed to dissociate rER‐LD and formed ectopic vesicle fusions

We next used transmission electron microscopy to observe the subcellular structures of *atvps13a* pollen after pollination. In the unhydrated phase immediately after pollination, no obvious structural defects were found in the mutant pollen (Fig. 3e). rER‐bound LDs, also observed in a previous study (Yamamoto *et al*., 2003), were abundant in both WT and *atvps13a* pollen grains (Fig. 3f). At 10 min after pollination, 47.0 ± 5.9% (mean ± SE) of the LDs had dissociated from the rER in WT pollen (Fig. 3f,g), whereas dissociation was rarely observed in the *atvps13a* mutant (1.0 ± 1.0%). At 20 min after pollination, the WT pollen grains formed enlarged vacuoles on the side opposite from the germination site, while such structure was not found in any of the mutant pollen (Fig. 3h).

Pollen grains are enriched in small Golgi‐derived vesicles (Yamamoto *et al*., 2003), and we found putative vesicle‐fused structures near the plasma membrane in the *atvps13a* mutant (indicated by arrows in Fig. 3f,h). These structures were surrounded by Golgi‐derived vesicles with a similar electron‐dense property, suggesting they were formed through aberrant fusions of these vesicles (Fig. S8). These observations indicate that AtVPS13a may be required to regulate organelle reorganization during pollen germination.

### AtVPS13a polarizes to future germination site and pollen tube clear zone

**Fig. 4:**
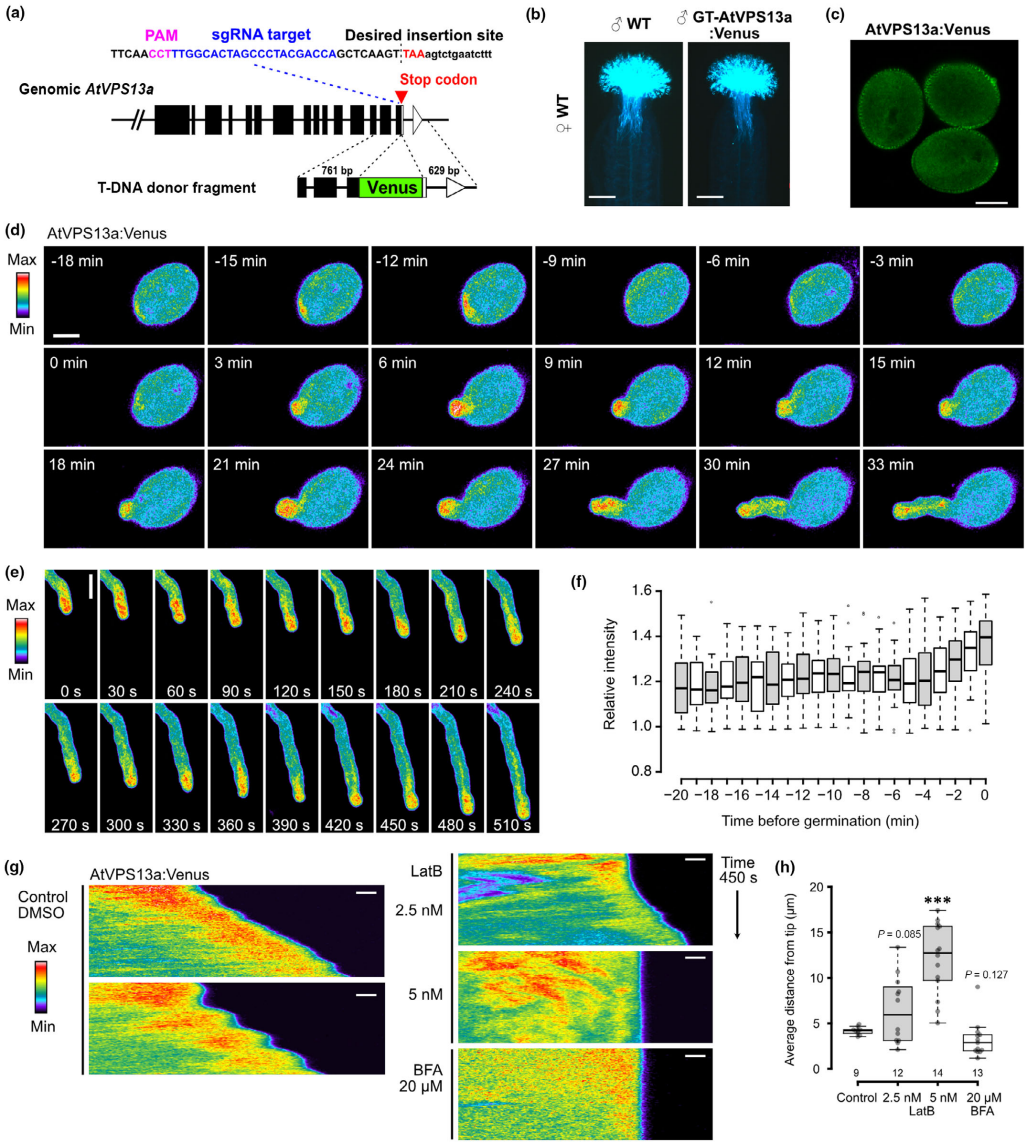
AtVPS13a:Venus polarized to future germination site and maintained at pollen tube tip by actin filament network. (a) CRISPR/Cas9‐mediated gene targeting of *Arabidopsis thaliana VPS13a*. The sgRNA target and protospacer adjacent motif (PAM) sequence near the stop codon are shown. A vector containing a donor fragment with homology regions to the desired insertion site and sgRNA‐expressing cassette was transferred into Cas9‐expressing *Arabidopsis*. (b) Aniline blue staining of pistils after pollination with WT or homozygous T_3_ GT‐AtVPS13a:Venus pollen for 3 h. Bars, 200 μm. (c) Pollen grains from homozygous T_3_ GT‐AtVPS13a:Venus plant excited by 514 nm laser. The signal from the pollen exine resulted from autofluorescence of the pollen wall. Bar, 10 μm. (d, e) Representative time‐lapse images of *in vitro* pollen germination of GT‐AtVPS13a:Venus pollen, AtVPS13a:Venus signal increased at the future germination site of germinating pollen (d) and pollen tube tip of germinated pollen (d, e). Bars, 10 μm. (f) Box plot of relative Venus fluorescence intensity of the average signal in a 5 μm diameter circle below future germination site against the average signal of the whole pollen grain, *n* = 19 pollen grains. (g) Representative kymographs of pollen tubes generated from time‐lapse imaging at 3‐s intervals for 450 s. Time‐lapse was captured from 10 to 30 min after LatB treatment or 30 to 60 min after BFA treatment. Bars, 2 μm. (h) Box plot of the average distance of peak Venus signal from pollen tube tip, the number below each box plot represents the number of pollen tube (*n*) analyzed; *P*‐value calculated from two‐tailed Student's *t*‐test; ***, *P* < 0.001. For box plots, the center line shows the median, box limits indicate the 25^th^ and 75^th^ percentiles, and whiskers extend 1.5 times the interquartile range from the 25^th^ and 75^th^ percentiles.

To observe AtVPS13a during pollen germination, we used CRISPR/Cas9‐mediated GT to tag the fluorescent protein Venus before the stop codon of the genomic *AtVPS13a* (Fig. 4a). The homozygous GT‐line (GT‐AtVPS13a:Venus) showed unaffected pollen germination on stigmas, suggesting that the fusion protein retained its function in pollen grains (Fig. 4b). AtVPS13a:Venus fluorescence was evenly distributed in a mature pollen grain before pollination (Fig. 4c). Live‐imaging of GT‐AtVPS13a:Venus pollen germination *in vitro* showed that AtVPS13a:Venus intensified at the future germination site and later focused in the elongating pollen tube tip region (Fig. 4d–f; Video S1). These results indicate that AtVPS13a participates in cellular polarization during pollen germination.

The establishment of cell polarity for pollen germination and tube growth relies on the regulated arrangement of F‐actin (Wu *et al*., 2010; Zhu *et al*., 2017; Liu *et al*., 2018). The actin fringe maintained in the subapical region of the pollen tube restricts large organelles from entering the apical clear zone, allowing a high concentration of small vesicles at the pollen tube tip (Kroeger *et al*., 2009; Cai *et al*., 2015). We used pharmacological approach to interfere with pollen tube F‐actin and study AtVPS13a:Venus localization in a pollen tube. Under control condition, AtVPS13a:Venus was focused at the pollen tube tip region, with the peak of averaged fluorescence positioned at 4.17 ± 0.44 μm (mean ± SD) from the pollen tube tip (Fig. 4g,h). During oscillatory growth, AtVPS13a:Venus signal appeared closer to the tip when the pollen tube actively elongated (Fig. 4g). The tip localization of AtVPS13a:Venus was visibly affected when 2.5 nM latrunculin B (LatB), an actin polymerization inhibitor, was applied to the growing pollen tube (6.43 ± 3.68 μm). At 5 nM LatB, AtVPS13a:Venus tip localization was completely disrupted (12.10 ± 3.96 μm, Fig. 4g,h). By contrast, 20 μM Brefeldin A (BFA) which inhibits vesicle trafficking and also indirectly affects F‐actin tended to shift AtVPS13a:Venus peak position closer to the tip (Fig. 4g,h). Bright‐field observation showed that BFA treatment shortened the clear zone at the pollen tube apex, while the clear zone was abolished in the 5 nM LatB‐treated samples (Fig. S9). These findings suggest that the spatio‐temporal distribution of AtVPS13a is correlated with the vesicle‐dense pollen tube clear zone.

We also found that AtVPS13a may be involved in vesicle secretion rather than endocytosis, as indicated by its different tip‐focusing pattern compared with the endocytic vesicle marker dye, FM4‐64 (Video S2).

### AtVPS13a co‐localizes with the vesicle secretion system

**Fig. 5:**
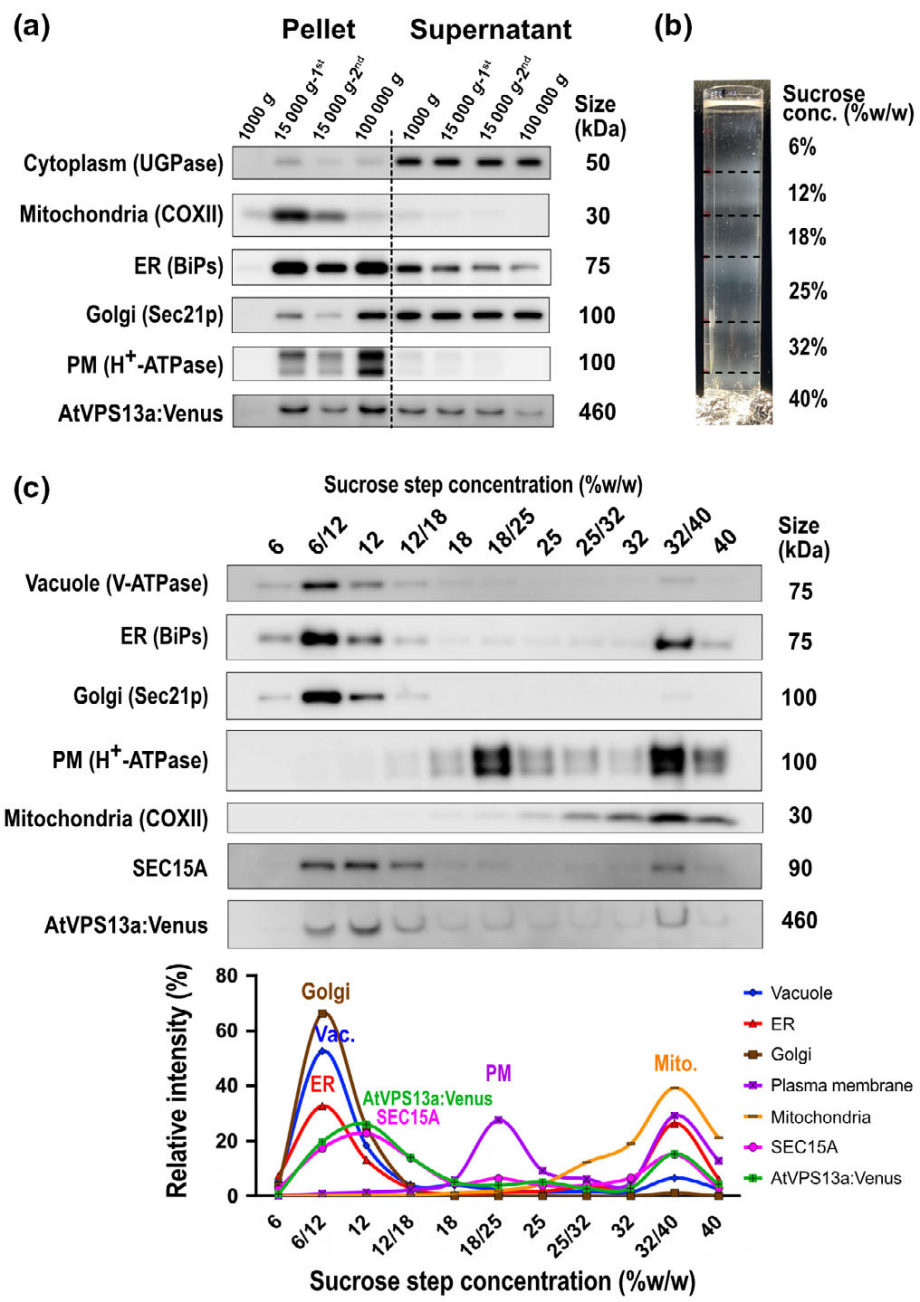
Identification of AtVPS13a:Venus coenriched proteins by proteomics. (a) Separation of organelles by differential centrifugation. Antibodies against various organelle markers are used to visualize organelle distribution in each fraction. An antibody against GFP is used to detect *Arabidopsis thaliana* VPS13a:Venus. The fractionation was independently performed three times using open flower samples collected on different days, with similar results obtained from each replicate. (b) Separation of the 100 000 ***g*** pellet of gene targeting‐VPS13a:Venus pollen protein by sucrose step gradient centrifugation. (c) Western blot analysis of each fraction from sucrose step gradient centrifugation to analyze the distribution patterns of organelle markers, AtVPS13a:Venus, and the exocyst subunit SEC15A. Line graphs represent the quantitative distribution of each component calculated by band intensity. ER, endoplasmic reticulum; PM, plasma membrane.

To gain insight into the molecular localization of AtVPS13a, we used a combination of biochemical analyses. Crude flower extract of the GT‐VPS13a:Venus line was fractionated by differential centrifugation. The AtVPS13a:Venus fusion protein was most enriched in the final 100 000 ***g*** pellet, which presumably contained microsomes (Fig. 5a). Thus, we further fractionated the microsome fraction isolated from mature pollen of the GT‐AtVPS13a:Venus line by sucrose step gradient centrifugation (Fig. 5b). AtVPS13a:Venus was most enriched in the 12% sucrose fraction, but exhibited a different distribution pattern from all other organelles initially investigated (Fig. 5c).

To identify cell components that co‐migrated with AtVPS13a:Venus, four sucrose fractions (6/12%, 12%, 12/18%, and 18/25%) were subjected to proteomic analysis by LC‐MS/MS. Out of 3046 detected proteins, the distribution pattern of 340 proteins showed a correlation with AtVPS13a‐Venus (Pearson's correlation coefficient > 0.8; Table S3). Gene Ontology (GO) enrichment analysis of these 340 proteins using PANTHER (Mi *et al*., 2019) identified proteins that belong to ‘Vesicle transport along actin filament’, ‘Golgi to plasma membrane transport’ or ‘exocytosis’ co‐migrated with AtVPS13a:Venus (Table S4). In agreement with the proteomic analysis, the distribution pattern of an exocyst subunit SEC15A, investigated by its specific antibody, strongly resembled that of AtVPS13a:Venus (Fig. 5c).

We also performed co‐immunopurification (co‐IP) of crude pollen proteins from the GT‐AtVPS13a:Venus line. In total, 82 proteins were uniquely detected in the AtVPS13a:Venus co‐IP fraction but were not detected in that of WT (Table S5). GO analysis showed that ‘exocytosis’ was the most significantly enriched term (*P* = 3.32e‐03) among the 82 proteins. Notably, exocyst complex subunits SEC8 and SEC5B, which are required for exocytosis during pollen germination (Hála *et al*., 2008), and Armadillo Repeat Only1 (ARO1), involved in polar cell growth (Gebert *et al*., 2008), co‐localized with AtVPS13a in both sucrose gradient purification and co‐IP (Tables S3, S5).

### AtVPS13a synchronizes with vesicle polarization following local Ca^2+^ signal

**Fig. 6:**
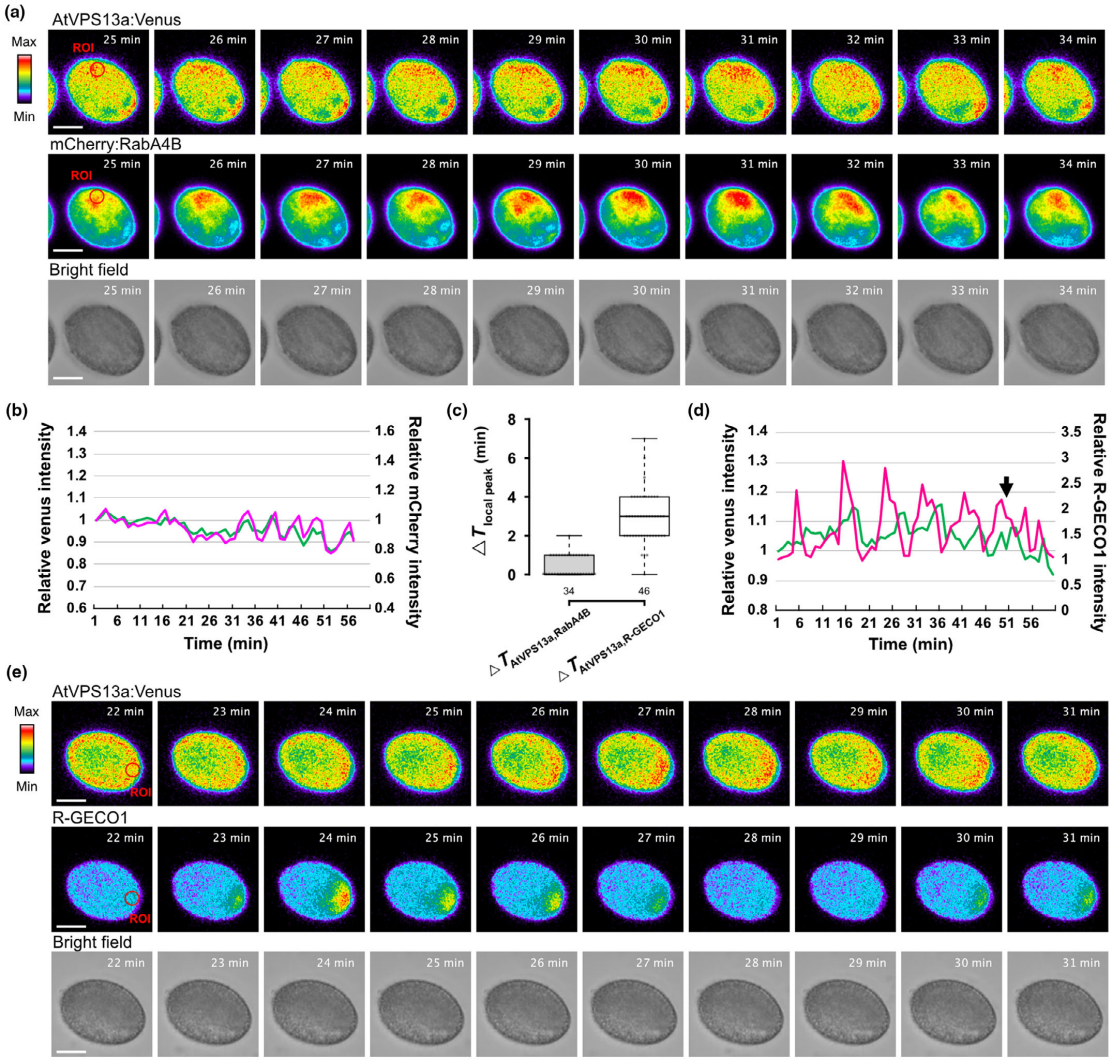
Ca^2+^ signaling preceded AtVPS13a:Venus and mCherry:RabA4B vesicle polarization in activated pollen grains. (a) Representative time‐lapse images of *in vitro* pollen germination of the dual reporter, *Arabidopsis thaliana* VPS13a:Venus and mCherry:RabA4B pollen. Region of interests (ROIs) indicate regions of interest for relative intensity calculation in (b). Bars, 10 μm. (b) The dynamic of the relative intensity of AtVPS13a:Venus (green) and mCherry:RabA4B (magenta) at the polarized site (ROI). (c) Box plot of the time difference between each oscillating fluorescence peak of AtVPS13a and RabA4B or R‐GECO1 during *in vitro* polarization. The center line shows the median, box limits indicate the 25^th^ and 75^th^ percentiles, and whiskers extend 1.5 times the interquartile range from the 25^th^ and 75^th^ percentiles. (d) The dynamic of the relative intensity of AtVPS13a:Venus (green) and R‐GECO1 (magenta‐red) at the polarized site (ROI). The arrow indicates the germination of the pollen grain. (e) Representative time‐lapse images of *in vitro* pollen germination of the dual reporter, AtVPS13a:Venus and R‐GECO1 pollen grain. Bars, 10 μm.

During pollen germination, polarized vesicle secretion and Ca^2+^ gradient occur at the future germination site (Iwano *et al*., 2004; Hoedemaekers *et al*., 2015). As mentioned in the previous section, AtVPS13a co‐localizes with vesicle‐related systems. Therefore, we investigated whether the distribution dynamics of AtVPS13a correlates with secretory vesicle movement or Ca^2+^ gradient during pollen germination. We introduced the secretory vesicle marker mCherry:RabA4B or the Ca^2+^ reporter R‐GECO1 into the GT‐AtVPS13a:Venus plant background to generate dual reporter lines. Interestingly, simultaneous live imaging of secretory vesicles and AtVPS13a:Venus during *in vitro* germination revealed that both signals accumulated at the polarized site with similar timing (Figs 6a–c, S10a; Video S3). However, each local peak of AtVPS13a:Venus occurred 2.74 ± 0.739 min after a Ca^2+^ peak at the polarized site (Figs 6c–e, S10b). These results demonstrate that AtVPS13a and RabA4B vesicle marker had synchronized local accumulation following a Ca^2+^ elevation at the future pollen germination site.

We captured a putative polarization initiation in pollen, supporting the association between increased Ca^2+^, AtVPS13a accumulation, and vesicle secretion. Notably, the background signal of AtVPS13a:Venus remained relatively stable in a prepolarized grain, but was distinctly perturbed by the first Ca^2+^ spike during polarization (Fig. S10c). Furthermore, during polarization, polarizing pollen secretes cell wall carbohydrates toward the future germination site, forming a germination plaque (Hoedemaekers *et al*., 2015; Li *et al*., 2017). We observed a spatial buildup between the outer pollen wall and the cytoplasm, coinciding with the Ca^2+^ and AtVPS13a:Venus waves during pollen polarization (Video S4). These phenotypes provide strong evidence that AtVPS13a follows Ca^2+^ signaling and mediates vesicle secretion.

### AtVPS13a mediates vesicle polarization during pollen germination

**Fig. 7:**
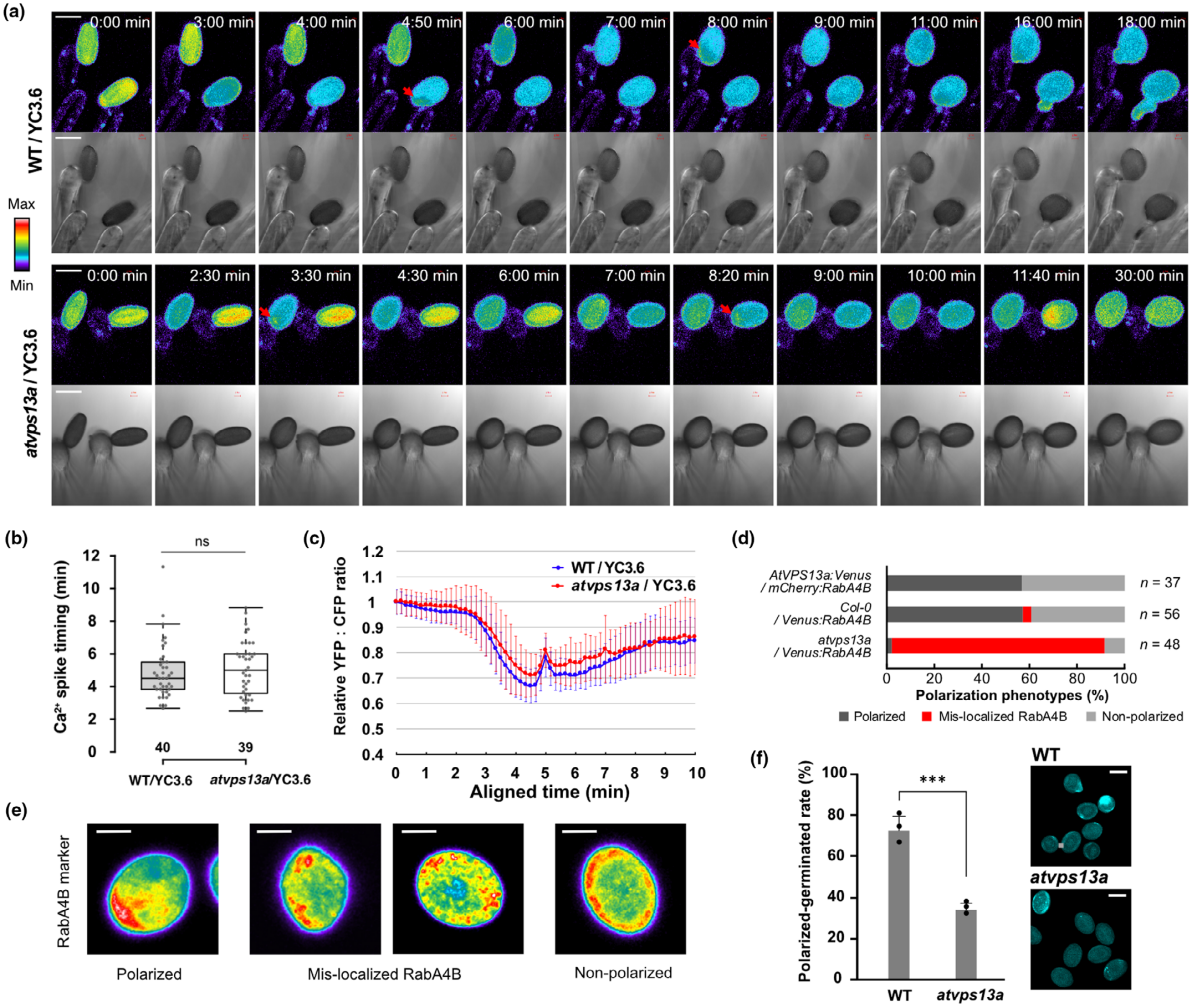
AtVPS13a mediates pollen polarization downstream of Ca^2+^ signaling. (a) Ratiometric images of adjusted YFP : CFP ratio from YC3.6 representing Ca^2+^ levels in Wild‐type (WT) *Arabidopsis thaliana* and *atvps13a* plant backgrounds. Red arrows indicate the first Ca^2+^ spike during pollen hydration in both WT and mutant pollen grains. Bars, 20 μm. (b) Box plot showing the timing of the first Ca^2+^ spike after pollen hydration initiation. The center line shows the median, box limits indicate the 25^th^ and 75^th^ percentiles, and whiskers extend 1.5 times the interquartile range from the 25^th^ and 75^th^ percentiles. The number below each box plot represents the number of pollen grains observed (*n*); ‘ns’ indicates nonsignificant difference between means of the two groups by two‐tailed Student's *t*‐test (*P* = 0.559). (c) Pollen Ca^2+^ dynamic at the papilla cell contact represented by ratiometric images of YFP : CFP of YC3.6. Time after pollination was aligned in the manner that makes the first Ca^2+^ spike appear at 5 min. (d) Percentage of pollen polarization phenotypes observed during *in vitro* pollen germination experiments, and (e) representative images of RabA4B localization for each phenotype. Bars, 10 μm. (f) Sum of polarized pollen (marked by callose deposition at pollen wall) and germinated pollen rate after short incubation in liquid pollen germination medium and stained with aniline blue. Data and error bars are mean ± SD. Asterisks indicate significant differences by two‐tailed Student's *t*‐test: ***, *P* < 0.001. Bars, 20 μm.

It is known that Ca^2+^ gradient forms within pollen grains, most concentrated at the papilla cell contact site after pollen hydration (Iwano *et al*., 2004). We investigated the local Ca^2+^ increase inside the pollinated pollen grain using the Ca^2+^ sensor Yellow Cameleon 3.6 (YC3.6). Consistent with the previous report, the cytoplasmic Ca^2+^ level rapidly decreased during pollen hydration (Iwano *et al*., 2004; Fig. 7a). We further found that the first Ca^2+^ spike appeared only briefly (< 30 s, Video S5) at 4.87 ± 1.70 min (mean ± SD) after hydration initiation in the WT (Fig. 7b). The *atvps13a*/YC3.6 pollen also showed a Ca^2+^ spike at the same timing (5.10 ± 1.69 min; two‐tailed Student's *t*‐test, *P* = 0.559). After the dissipation of the first Ca^2+^ spike, a gradual cytoplasmic Ca^2+^ increase until germination was observed (Fig. 7c; Video S5). This Ca^2+^ dynamic was similarly observed in both WT and *atvps13a* pollen grains (Fig. 7a–c), suggesting that AtVPS13a is not required to trigger this signal but functions to establish pollen polarity downstream of the Ca^2+^ gradient.

AtVPS13a has a predicted Ca^2+^‐dependent lipid‐binding C2 domain (Fig. 2b), which may mediate Ca^2+^ signaling response during pollen germination. We found that an artificial Ca^2+^ increase by calcium ionophore treatment in *Arabidopsis* mesophyll protoplast led to the re‐localization of mNeonGreen:C2_VPS13a_ to the membrane‐enriched fraction (Fig. S11a). Using CRISPR/Cas9, we generated an *AtVPS13a* mutant lacking the complete C2 domain and found that the pollen tube tip focusing of AtVPS13aΔC2:Venus was partially lost, while the pollen tube clear zone was maintained (Fig. S11b–e). The result indicates that the C2 domain is required to regulate the cellular distribution of AtVPS13a. The pollen germination rate and tube growth were also affected in the C2 deletion lines (Fig. S11c,f). This may result from the combined effect of reduced polarity and protein stability, as *AtVPS13aΔC2:Venus* pollen accumulated a reduced amount of the fusion protein (Fig. S11g).

During pollen polarization, polarized vesicle secretion contributes to the formation of the germination plaque, marked by the deposition of callose and other cell wall polysaccharides at the site where the pollen tube will emerge (Hoedemaekers *et al*., 2015; Li *et al*., 2017). Based on our observations of AtVPS13a co‐accumulating with secretory vesicles in polarizing pollen (Fig. 6a,b), we hypothesized that AtVPS13a plays a role in vesicle trafficking that supports cell wall secretion. Time‐lapse imaging during *in vitro* germination revealed that RabA4B vesicles became more static and mis‐localized in most of the mutant pollen (Fig. 7d,e; Video S6). Additionally, RabA4b vesicles were mis‐localized in *atvps13a* pollen tubes. The apical concentration of RabA4B in these tubes was noticeably disrupted, appearing more diffuse or, in some cases, remaining confined within the pollen grain rather than localizing to the tip (Fig. S12). We also found that the rate of callose deposition on the pollen wall decreased in the mutant. Pollen grains were incubated in liquid PGM and stained with aniline blue to observe callose deposited onto the pollen cell wall. Our results showed that at least one callose spot was present in 72.6 ± 6.90% (mean ± SD) of WT pollen but only 33.8 ± 2.90% in *atvps13a* pollen (Fig. 7f).

Taken together, our findings suggest that AtVPS13a functions downstream of calcium signals in pollen polarization. The signal may alter protein‐membrane binding through its C2 domain. Proper localization of AtVPS13a is essential for maintaining vesicle trafficking to the polarized site, ensuring sufficient secretion for successful pollen germination.

## Discussion

In this study, we rediscovered an overlooked phenomenon as an essential step toward successful pollen germination. In *Brassica oleracea*, LDs were found unwinded from ER in hydrating pollen grains (Elleman & Dickinson, 1986). We found that this process required the function of AtVPS13a in *Arabidopsis* pollen because, in hydrated *atvps13a* pollen, LDs remained tightly bound to the rER (Fig. 3f,g). We speculate that AtVPS13a‐mediated LD release is necessary to expose their surface for further catabolism. Some reports indicate active consumption (Zienkiewicz *et al*., 2010, 2012) and structural protein degradation (Kretzschmar *et al*., 2018) of LDs in pollen. It is possible that AtVPS13a‐mediated LD release is a prerequisite that primes the pollen grain for polar growth.

Since we found that AtVPS13a biochemically co‐localized with the exocyst subunits (SEC5B and SEC8), our study suggests that AtVPS13a may be functionally related to exocytosis. The exocyst complex comprises subcomplexes that dynamically bind cytoplasmic secretory vesicles (Ahmed *et al*., 2018). The secretory vesicle is then tethered to the destined membrane by SEC3 and EXO70 subunits before subsequent membrane fusion (Ahmed *et al*., 2018; Mei & Guo, 2018). We found that AtVPS13a and RabA4B secretory vesicle marker synchronized their accumulation at the germination site (Fig. 6a–c), where exocytosis mediates cell wall deposition (Hála *et al*., 2008; Bloch *et al*., 2016; Li *et al*., 2017). Furthermore, *atvps13a* pollen formed exocytotic vesicle fusion structures in hydrated pollen grains (Fig. S8), indicating that AtVPS13a is required to prevent ectopic fusion of these vesicles.

We also found that AtVPS13a co‐localizes with a member of the ARO subfamily proteins, recently reported to have a scaffolding function to limit ROP signaling to the polar growth sites (Kulich *et al*., 2020). Pollen ARO1, which biochemically co‐localized with AtVPS13a (Tables S3, S5), localizes to the future germination site (Vogler *et al*., 2015), and is required for tip growth maintenance by regulating the F‐actin network (Gebert *et al*., 2008). It is possible that AtVPS13a is needed to establish polarized exocytosis by cooperating with the exocyst complex and/or ARO1.

After pollination, pollen uptake water and extracellular Ca^2+^ through the contact with the papilla cell, which may provide directional cues for the pollen tube emergence site (Lush *et al*., 1998; Iwano *et al*., 2004; Swanson *et al*., 2004). We discovered that pollen briefly uptakes extracellular Ca^2+^ from the papilla contact site before hydration completion (Fig. 7a,b). To our knowledge, this study is the first to visualize this brief Ca^2+^ uptake as early as 5 min after hydration initiation. AtVPS13a and RabA4B may be recruited to this Ca^2+^ elevated site (Fig. 6a–e).

Interestingly, AtVPS13a is the primary *Arabidopsis* VPS13 important for pollen germination and the only homolog predicted to contain a C2 domain. When the Ca^2+^ level was artificially elevated using a calcium ionophore, the C2 domain expressed in mesophyll protoplasts became more enriched in the membrane fraction (Fig. S11a). Our data also showed that the C2 domain is required for proper AtVPS13a polarization at the tip of the pollen tube (Fig. S10b,c). This is consistent with the observation that the highest concentration of Ca^2+^ is maintained at the tip during pollen tube growth (Holdaway‐Clarke *et al*., 1997; Iwano *et al*., 2009), and suggests that the C2 domain may play a role in the Ca^2+^‐dependent membrane localization of AtVPS13a at the tip, as found in other studies (Corbalan‐Garcia & Gómez‐Fernández, 2014). It should be noted that LatB treatment disrupts both F‐actin and the pollen tube tip Ca^2+^ gradient in lily pollen (Cárdenas *et al*., 2008). Therefore, the possibility that the disruption of AtVPS13a:Venus tip focusing by LatB (Fig. 4g,h) is caused by the perturbation of Ca^2+^ gradient cannot be ruled out.

The pattern of AtVPS13a:Venus signal in polarizing pollen grains appears less distinct compared with the highly polarized RabA4B vesicle and local Ca^2+^ peaks (Fig. 6a,e). This could be because AtVPS13a potentially localizes to multiple organelles and cellular locations, as has been reported in VPS13 homologs (Bean *et al*., 2018; Kumar *et al*., 2018; Dziurdzik & Conibear, 2021; Leonzino *et al*., 2021). Therefore, only a fraction of AtVPS13a may participate in pollen polarization, making its co‐accumulation with vesicles more apparent at the polarized site. Additionally, AtVPS13a may not be the sole regulator of pollen polarization, as pollen lacking AtVPS13a still showed significantly reduced but detectable polarization (Fig. 7d,e).

RAC/ROP signaling and its downstream pathways are good candidates for triggering pollen polarization, as pollen hydration in an appropriate medium is enough to activate *Nicotiana tabacum* RAC (Chen *et al*., 2013). AtVPS13a likely acts as a positive regulator after initial pollen activation to ensure correct vesicle trafficking to the polarized site, providing sufficient vesicle secretion for pollen germination.

Recent studies found that VPS13 proteins tether or transport lipid between organelles (Li *et al*., 2020; Cai *et al*., 2022). They localize to various organelle contact sites depending on cell type and cellular context (Park & Neiman, 2012; Kumar *et al*., 2018; Kolakowski *et al*., 2021; Wang *et al*., 2021). AtVPS13a contained hydrophobic cavity at its N‐terminus that may transfer lipids (Li *et al*., 2020; Cai *et al*., 2022) and an amphipathic helix at its C‐terminus that may anchor it to a biological membrane (Kumar *et al*., 2018; Fig. S3a,b), similar to other VPS13 proteins. Although speculative, it is conceivable that AtVPS13a may mobilize lipid from a source, such as free LDs, to other organelles either directly or through an intermediate membrane transport system such as vesicles. Potential sink organelles include extending membranes such as the vacuole or the rapidly expanding plasma membrane.

## Accession numbers

*DIC2* (At4g24570), *AtVPS13a* (At1g48090), *AtVPS13b* (At4g17140), *AtSHRUBBY* (At5g24740), and *AtRabA4B* (At4g39990).

## Competing interests

None declared.

## Author contributions

SF conceived the study. S Tangpranomkorn, SF and S Takayama planned the experiments. S Tangpranomkorn, SF and S Takayama wrote the manuscript with inputs from all other authors. S Tangpranomkorn conducted most of the experiments and data analysis. Y Kimura performed the scanning electron microscopy experiment, the protoplast experiment, and some parts of the pollen imaging experiments. MI helped establish the luciferase assay. FI performed the transmission electron microscopy experiments. Y Kato performed the phylogenetic analysis. TS conducted the bulk‐segregant sequence analysis. TN performed the pollen staining test.

## Supporting information


**Fig. S1** Luciferase‐based pollen compatibility assay.
**Fig. S2** Phenotypes of the *atvps13a* mutants isolated in the genetic screen.
**Fig. S3** Structural conservation of AtVPS13 proteins and their homologs.
**Fig. S4** Reduced male transmission efficiency of *atvps13a* allele.
**Fig. S5** *atvps13a* mutant pollen properly developed into mature pollen grains.
**Fig. S6** Genotype of genome‐edited *atvps13b* in Col‐0 background.
**Fig. S7** RNA‐seq counts from a Transcriptome variation analysis database (travadb.org).
**Fig. S8** Ectopic vesicle fusion in the hydrated *atvps13a* pollen grains.
**Fig. S9** AtVPS13a:Venus is maintained at pollen tube tip region and enriched at subapical clear zone.
**Fig. S10** Dynamic of relative intensity of AtVPS13a:Venus with mCherry:RabA4B, or R‐GECO1 at the polarized site in dual reporter pollen grains.
**Fig. S11** Putative C2 domain of AtVPS13a is important for efficient pollen germination and its distribution in pollen tube.
**Fig. S12** Mis‐localization of RabA4B vesicles in *atvps13a* pollen tubes.

**Table S1** List of oligonucleotides used in this study.
**Table S2** List of protein databases used to retrieve VPS13 sequence of each species.
**Table S3** List of detected proteins in selected sucrose fractions and their Pearson correlation coefficient with *Arabidopsis thaliana* VPS13a.
**Table S4** Result of PANTHER GO‐Slim Biological Process analysis of proteins with high (> 0.8) Pearson correlation coefficient with *Arabidopsis thaliana* VPS13a.
**Table S5** List of proteins uniquely found in *Arabidopsis thaliana* VPS13a:Venus Co‐IP sample and their Pearson correlation coefficients with AtVPS13a:Venus in sucrose gradient fractions proteomics.

**Video S1** Live imaging of *in vitro* pollen germination of gene targeting AtVPS13a:Venus pollen.

**Video S2** Live imaging of *in vitro* pollen tube growth of AtVPS13a:Venus and endocytic vesicle marker FM4‐64 dye.

**Video S3** Live imaging of *in vitro* pollen germination of dual reporter line gene targeting AtVPS13a:Venus and mCherry:RabA4B.

**Video S4** Live imaging of *in vitro* pollen germination of dual reporter line gene targeting AtVPS13a:Venus and Ca^2+^ sensor R‐GECO1 showing AtVPS13a:Venus and Ca^2+^ relationship with cell wall deposition at the polarized site.

**Video S5** *In vivo* pollen Ca^2+^ spike during pollen hydration shown by ratiometric YFP/CFP video of pollen expressing YC3.6 calcium reporter.

**Video S6** Live imaging of *in vitro* pollen germination assay showing polarization of secretory vesicle marker RabA4B in *AtVPS13a* pollen grain and mis‐localized RabA4B signal in *atvps13a* pollen grain.
Please note: Wiley is not responsible for the content or functionality of any Supporting Information supplied by the authors. Any queries (other than missing material) should be directed to the *New Phytologist* Central Office.

## Data Availability

The data supporting the finding of this study are available in the main text and Supporting Information of this manuscript. The proteomic data of sucrose fractions and co‐immunopurification are available in Tables S3 and S5, respectively.
