# Supplementary material for: A land plant‐specific VPS13 mediates polarized vesicle trafficking in germinating pollen
Source: New Phytol. 2024 Dec 1;245(3):1072–89. doi: 10.1111/nph.20277 (PMC11712023; doi:10.1111/nph.20277)
Supplement: Supplementary file 1 — Fig. S1 Luciferase‐based pollen compatibility assay. Fig. S2 Phenotypes of the atvps13a mutants isolated in the genetic screen. Fig. S3 Structural conservation of AtVPS13 proteins and their homologs. Fig. S4 Reduced male transmission efficiency of atvps13a allele. Fig. S5 atvps13a mutant pollen properly developed into mature pollen grains. Fig. S6 Genotype of genome‐edited atvps13b in Col‐0 background. Fig. S7 RNA‐seq counts from a Transcriptome variation analysis database (travadb.org). Fig. S8 Ectopic vesicle fusion in the hydrated atvps13a pollen grains. Fig. S9 AtVPS13a:Venus is maintained at pollen tube tip region and enriched at subapical clear zone. Fig. S10 Dynamic of relative intensity of AtVPS13a:Venus with mCherry:RabA4B, or R‐GECO1 at the polarized site in dual reporter pollen grains. Fig. S11 Putative C2 domain of AtVPS13a is important for efficient pollen germination and its distribution in pollen tube. Fig. S12 Mis‐localization of RabA4B vesicles in atvps13a pollen tubes. [file NPH-245-1072-s003.pdf]

New Phytologist

New Phytologist Supporting Information

Article title: A land plant specific VPS13 mediates polarized vesicle trafficking in germinating pollen

Authors: Surachat Tangpranomkorn, Yuka Kimura, Motoko Igarashi, Fumiko Ishizuna, Yoshinobu Kato, Takamasa Suzuki, Takuya Nagae, Aya Yoshida, Sota Fujii*, Seiji Takayama*

Article acceptance date: 29 October 2024

The following Supporting Information is available for this article:

Fig. S1 Luciferase-based pollen compatibility assay

Fig. S2 Phenotypes of the *atvps13a* mutants isolated in the genetic screen

Fig. S3 Structural conservation of AtVPS13 proteins and their homologs

Fig. S4 Reduced male transmission efficiency of *atvps13a* allele

Fig. S5 *atvps13a* mutant pollen properly developed into mature pollen grains

Fig. S6 Genotype of genome-edited *atvps13b* in Col-0 background

Fig. S7 RNA-seq counts from a Transcriptome variation analysis database (travadb.org)

Fig. S8 Ectopic vesicle fusion in the hydrated *atvps13a* pollen grains

Fig. S9 AtVPS13a:Venus is maintained at pollen tube tip region and enriched at subapical clear zone

Fig. S10 Dynamic of relative intensity of AtVPS13a:Venus with mCherry:RabA4B, or R-GECO1 at the polarized site in dual reporter pollen grains

Fig. S11 Putative C2 domain of AtVPS13a is important for efficient pollen germination and its distribution in pollen tube

Fig. S12 Mis-localization of RabA4B vesicles in *atvps13a* pollen tubes

Table S1 List of oligonucleotides used in this study

Table S2 List of protein databases used to retrieve VPS13 sequence of each species

Table S3 List of detected proteins in selected sucrose fractions and their Pearson correlation coefficient with *Arabidopsis thaliana* VPS13a

Table S4 Result of PANTHER GO-Slim Biological Process analysis of proteins with high (>0.8) Pearson correlation coefficient with *Arabidopsis thaliana* VPS13a

Table S5 List of proteins uniquely found in *Arabidopsis thaliana* VPS13a:Venus Co-IP sample and their Pearson correlation coefficients with AtVPS13a:Venus in sucrose gradient fractions proteomics

Video S1 Live imaging of *in vitro* pollen germination of gene targeting AtVPS13a:Venus pollen

Video S2 Live imaging of *in vitro* pollen tube growth of AtVPS13a:Venus and endocytic vesicle marker FM4-64 dye

Video S3 Live imaging of *in vitro* pollen germination of dual reporter line gene targeting AtVPS13a:Venus and mCherry:RabA4B

Video S4 Live imaging of *in vitro* pollen germination of dual reporter line gene targeting AtVPS13a:Venus and Ca^{2+} sensor R-GECO1 showing AtVPS13a:Venus and Ca^{2+} relationship with cell wall deposition at the polarized site

Video S5 *In vivo* pollen Ca^{2+} spike during pollen hydration shown by ratiometric YFP/CFP video of pollen expressing YC3.6 calcium reporter

Video S6 Live imaging of *in vitro* pollen germination assay showing polarization of secretory vesicle marker RabA4B in *AtVPS13a* pollen grain and mis-localized RabA4B signal in *atvps13a* pollen grain

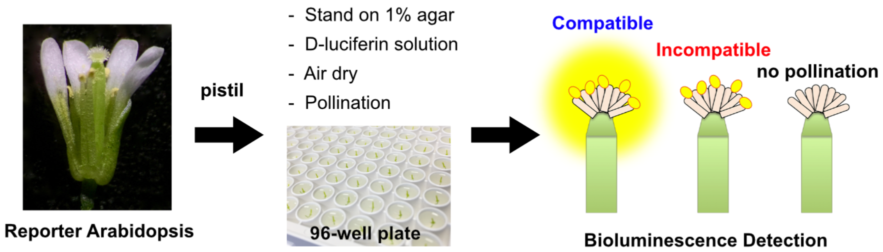

Fig. S1 Luciferase-based pollen compatibility assay. A pistil of an emasculated flower from the reporter *Arabidopsis* line was stood in each well of a 96 well plate containing solid agar. After allowing a small drop of luciferin to dry on the stigma, the pistil was subjected to pollination. Bioluminescence was detected on a microplate reader platform.

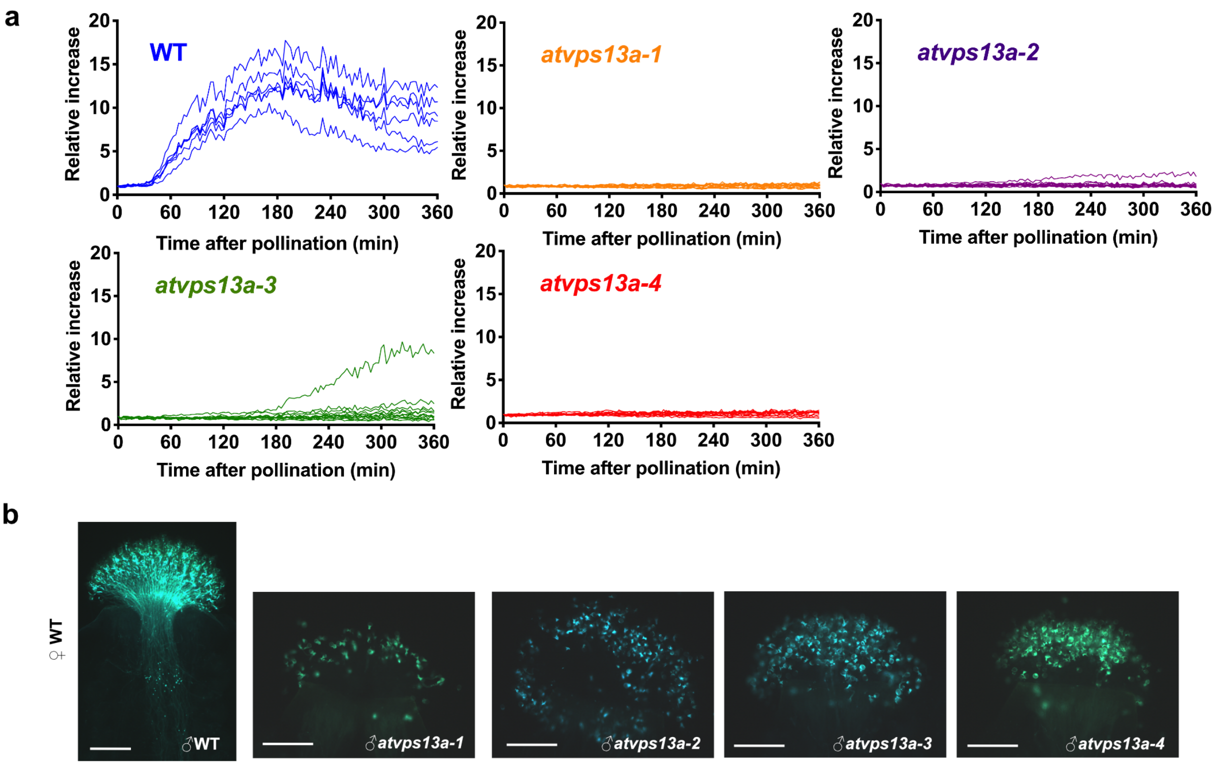

Fig. S2 Phenotypes of the *atvps13a* mutants isolated from the genetic screen. (**a**), Relative increases in luciferase activity in the reporter *Arabidopsis thaliana* pistils after pollination with wild-type (WT) and mutant *atvps13a1–4* pollen. (**b**), Representative aniline blue-stained images of pistils 6 h after pollination with WT or *atvps13a1–4* pollen. Scale bars: WT, 200 µm; *atvps13a1–4*, 100 µm.

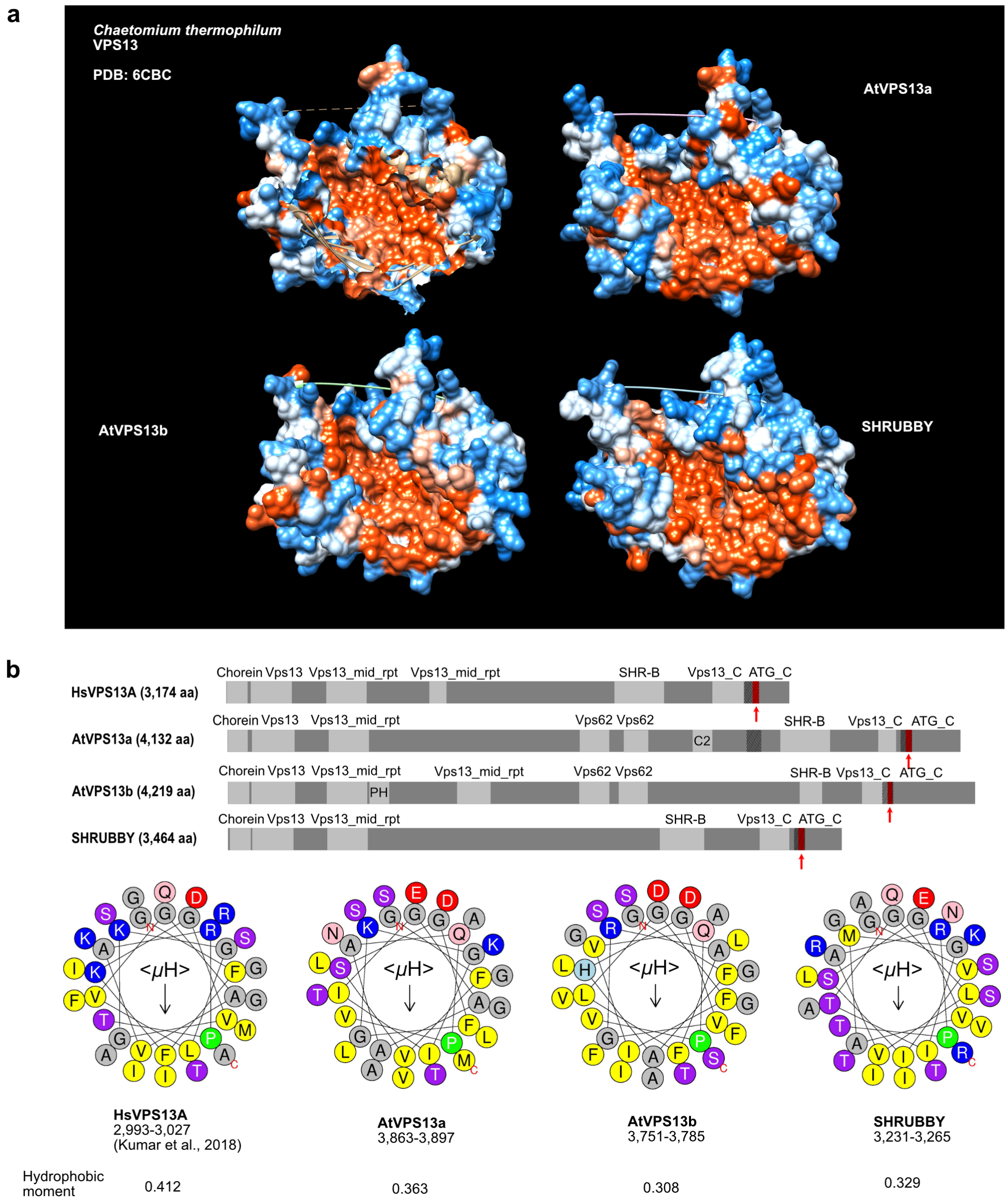

Fig. S3 Structural conservation of AtVPS13 proteins and their homologs. (**a**), *Arabidopsis thaliana* VPS13 models showing conservation of the hydrophobic cavity in the N-terminus. The 3D structure models of AtVPS13 were made using the Phyre2 homology-based modelling method (Kelly et al., 2015). The hydrophobicity surface colors by UCSF Chimera (Pettersen et al., 2004) are scaled from blue (most hydrophilic) to white to orange red (most hydrophobic) on the Kyte-Doolittle scale. (**b**), Comparison of the amphipathic helices in the ATG_C domains of HsVPS13A and AtVPS13. The locations of the helices are marked by red arrows. Helical wheel

projections by Heliquest (heliquest.ipmc.cnrs.fr; Gautier et al., 2008) show the vector of hydrophobic moment <μH> pointing toward the hydrophobic sides.

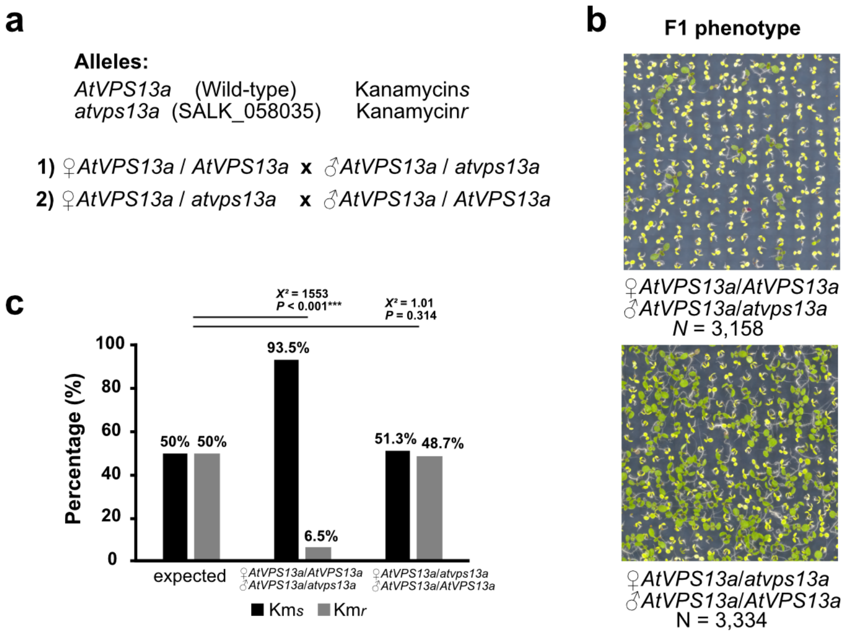

Fig. S4 Reduced male transmission efficiency of *atvps13a* allele. (**a**), *Arabidopsis thaliana* VPS13a alleles and genotypes of plants used in reciprocal cross experiment. (**b**), Representative image of F_1 seedling phenotype screened on 1/2MS + Kanamycin, F_1 seedling lacking *atvps13a*/Km^r allele could not grow well on selective plate. *N* equals to total number of seedlings scored in each crossing pair. (**c**), Percentage of F_1 phenotype from each crossing pair, *P*-value was calculated from Chi-squre (X^2) test for determining their difference from expected Mendelian's segregation frequency.

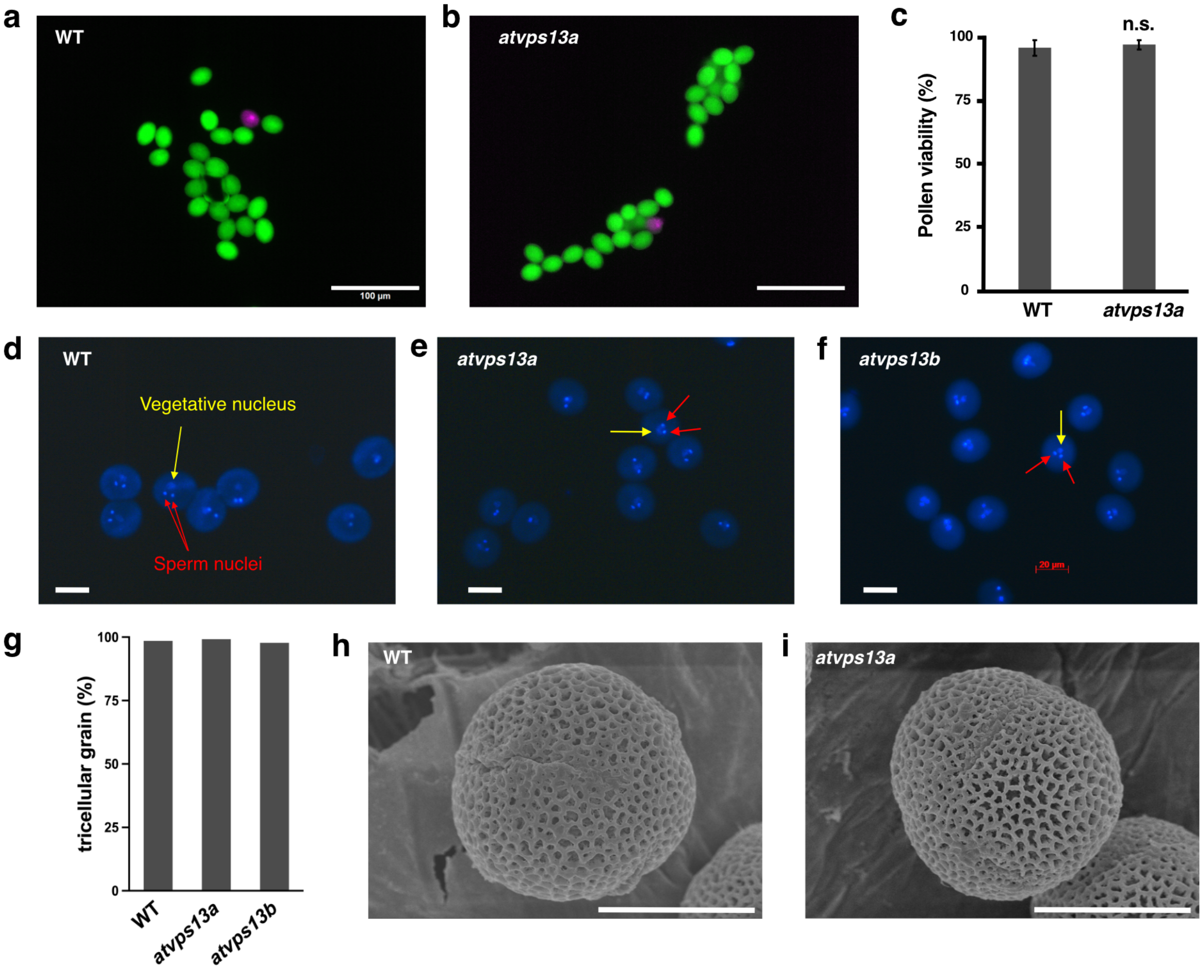

Fig. S5 *atvps13a* mutant pollen properly developed into mature pollen grains. Pollen viability assay by double FDA-PI staining of wild-type *Arabidopsis thaliana*, WT (**a**) and *atvps13a* (**b**) pollen grains. (**c**) Pollen viability of *atvps13a* was not different from WT, two-tailed Student's t-test, p = 0.445, n.s. not significant, mean ± SD. (**d-f**) DAPI staining of pollen grains from WT (**d**), *atvps13a* (**e**), and *atvps13b* (**f**) open flowers. (**g**), Percentages of tricellular pollen grains (n=205, 244, and 270, respectively). (**h, i**) Scanning electron microscopy of mature WT and *atvps13a* pollen, respectively. Scale bars, (**a,b**) = 100 µm, (**d-f**) = 20 µm, (**h,i**) = 10 µm.

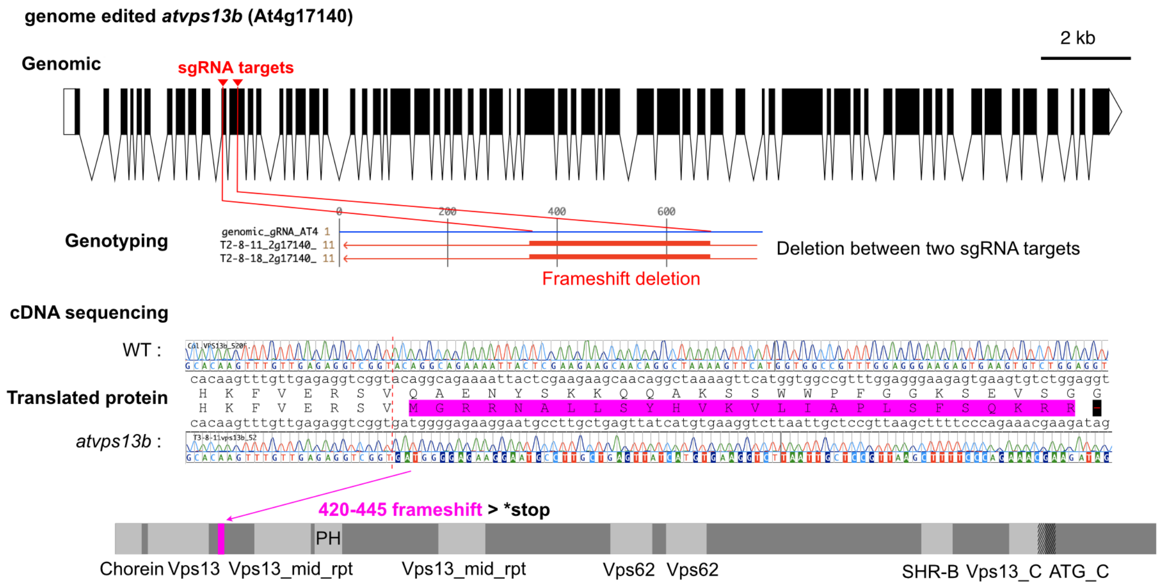

Fig. S6 Genotype of genome-edited *atvps13b* in Col-0 background. The alignment of the genomic region showed frameshift deletion occurred in genome-edited plant resulting in a premature stop codon after amino acid 445.

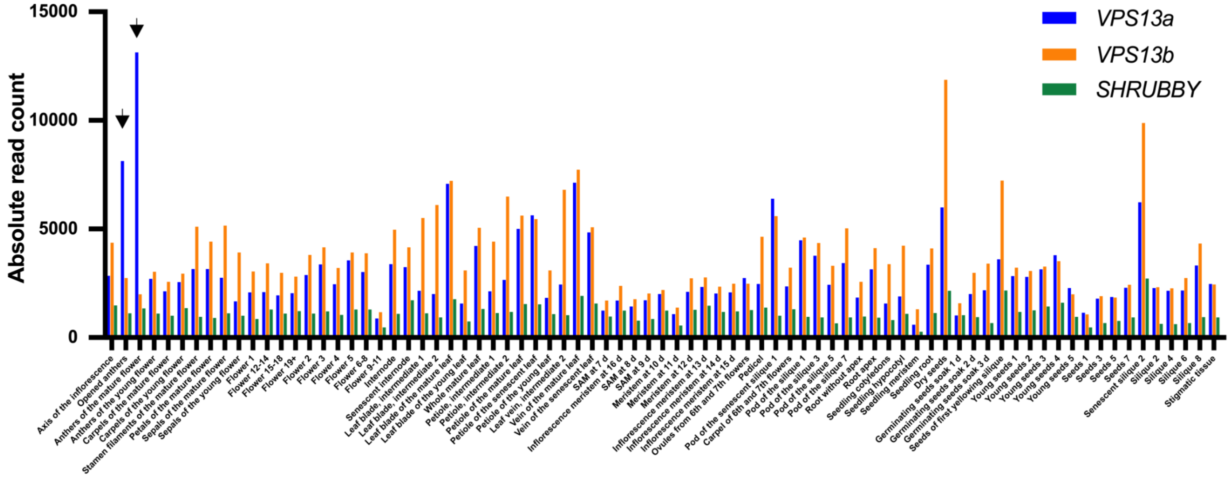

Fig. S7 RNA-seq counts from a Transcriptome variation analysis database (travadb.org; Klepikova et al., 2016). *Arabidopsis thaliana VPS13a* is expressed at higher levels than *AtVPS13b* and *SHRUBBY* in opened anthers and anthers of mature flowers (arrows).

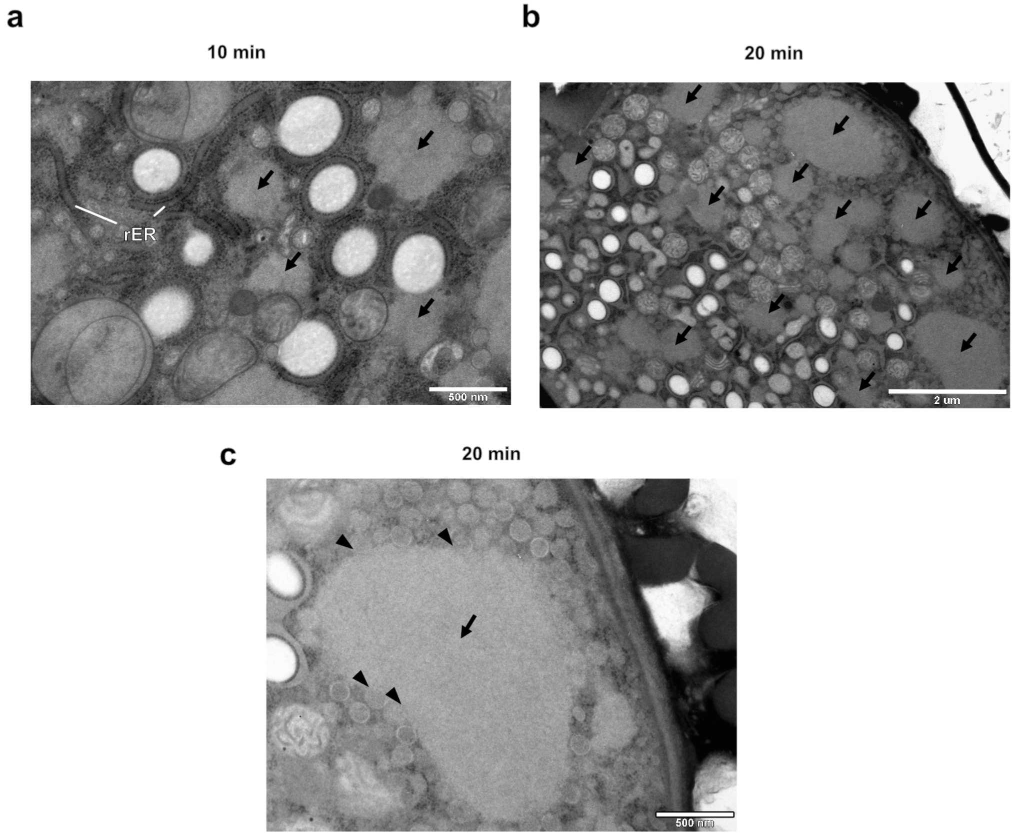

Fig. S8 Ectopic vesicle fusion in the hydrated *atvps13a* pollen grains. (**a**) and (**b**), Transmission electron micrographs of hydrated *Arabidopsis thaliana vps13a* pollen at 10 min (a) and 20 min (b) after pollination. (**c**), Higher magnification of *atvps13a* pollen at 20 min after pollination, putative vesicle fusion structure was surrounded by many small vesicles. Fusions of vesicles and the structure were observed (arrow heads). rER, rough endoplasmic reticulum; arrow, putative vesicle fusion structure; scale bars = 500 nm in (a) and (c), 2 µm in (b).

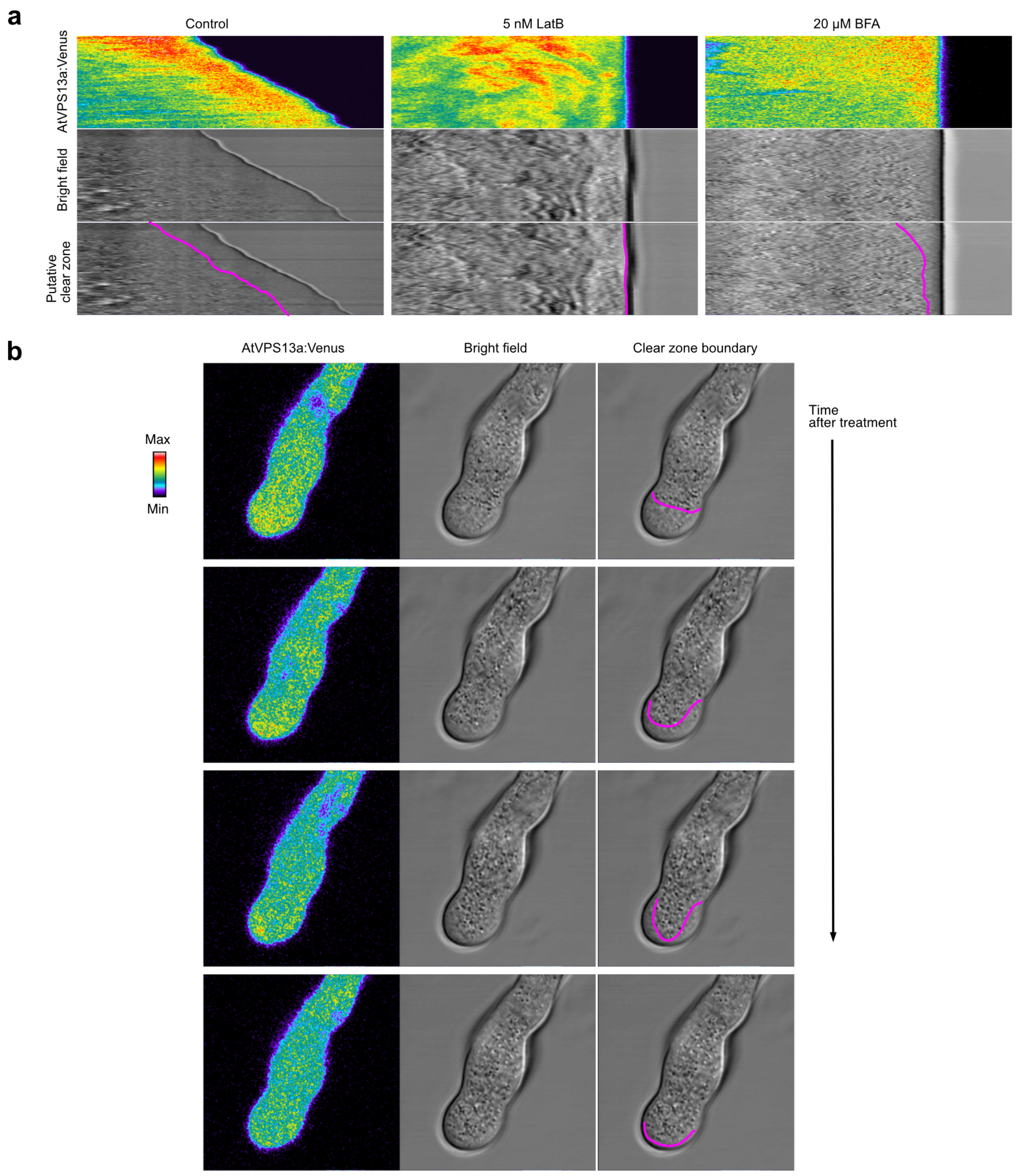

Fig. S9 AtVPS13a:Venus is maintained at pollen tube tip region and enriched at subapical clear zone. (**a**), Representative kymographs of *Arabidopsis thaliana* pollen tubes generated from time-lapse imaging captured after LatB or BFA treatment, scale bar = 2 µm. Putative clear zone boundary at pollen tube subapical region was drawn in magenta line. (**b**), representative

images of compromised clear zone after 20 µM BFA treatment showing spatio-temporal enrichment of AtVPS13a:Venus at the clear zone (magenta lines).

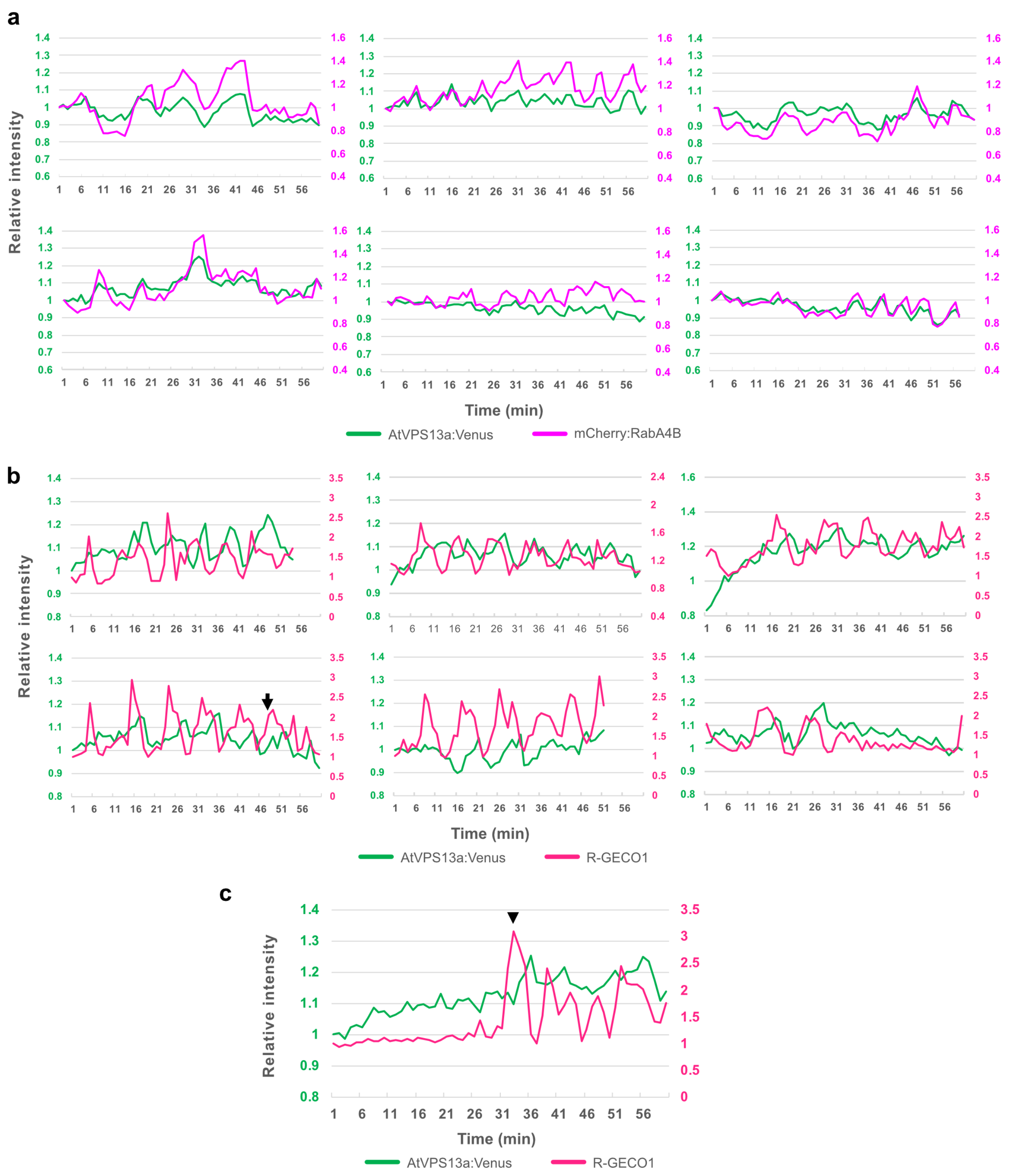

Fig. S10 Dynamic of relative intensity of AtVPS13a:Venus with mCherry:RabA4B, or R-GECO1 at the polarized site in dual reporter pollen grains. (**a**), Dynamic of relative intensity of *Arabidopsis thaliana* VPS13a:Venus (green) and mCherry:RabA4B (magenta) at the polarized site (5μm diameter). (**b**), Dynamic of relative intensity of AtVPS13a:Venus (green) and R-GECO1 (magenta-red) at the polarized site (5μm diameter). Left y-axis represents relative intensity of

AtVPS13a:Venus, Right y-axis represents relative intensity of mCherry:RabA4B (a) or R-GECO1 (b). Arrow indicates the germination of a pollen grain. (**c**), Dynamic of AtVPS13a:Venus intensity during transitioning to pollen polarization state. Accumulation of AtVPS13a:Venus at the polarized site increased soon following the first Ca^{2+} spike (arrow head).

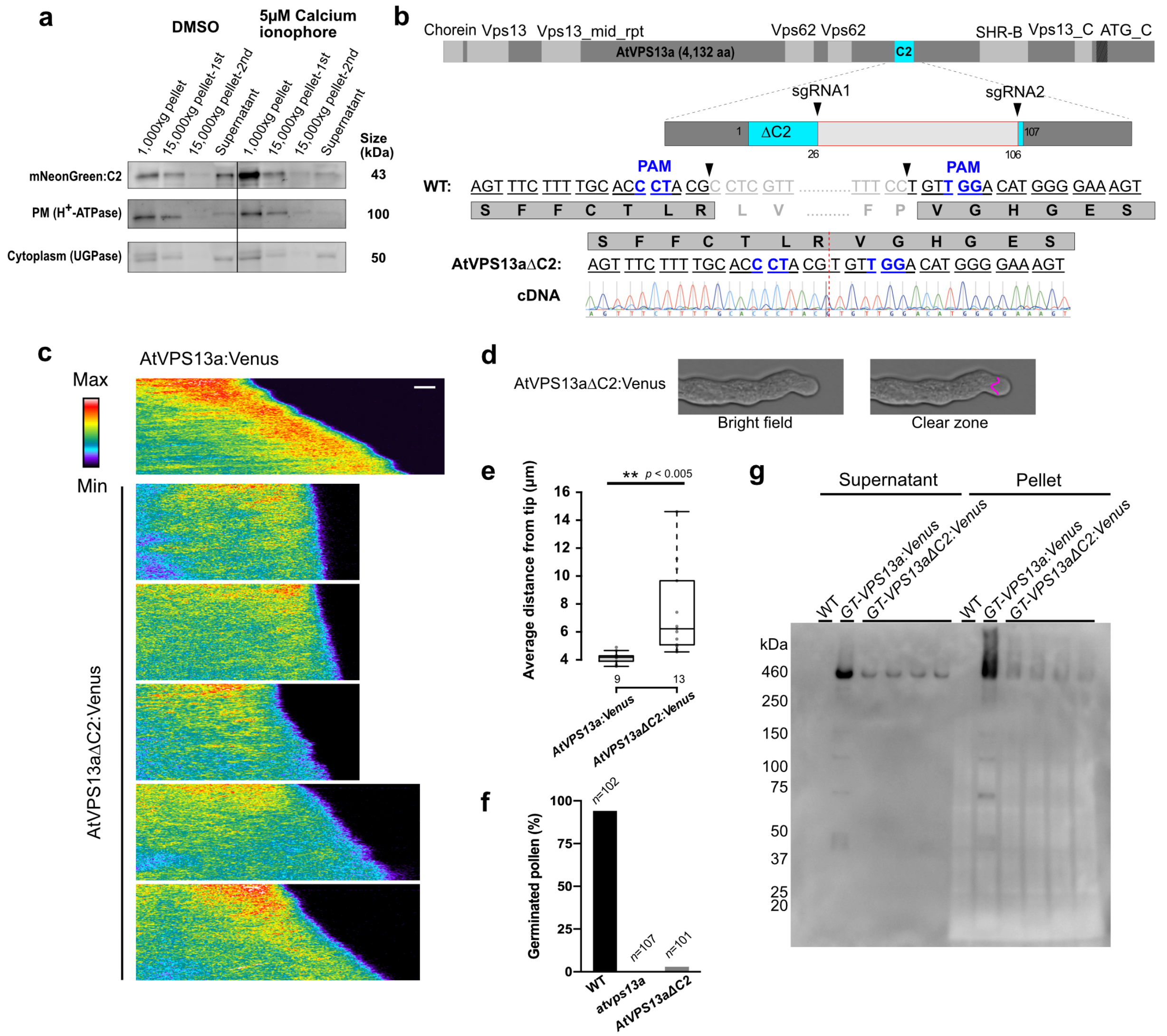

Fig. S11 Putative C2 domain of AtVPS13a is important for efficient pollen germination and its distribution in pollen tube. (**a**), Western blot analysis of fractionated *Arabidopsis thalaiana* mesophyll protoplast expressing mNeonGreen:C2$_{AtVPS13a}$ by differential centrifugation, the protoplast was treated with 5µM calcium ionophore for 10 min to artificially increase protoplast Ca^{2+} concentration, or DMSO as a control. mNeonGreen:C2$_{AtVPS13a}$ was more enriched in plasma membrane marker, H^+-ATPase, fraction after calcium ionophore treatment. (**b**), Generation of genome-edited *AtVPS13aΔC2.* Sites of the single guide RNAs (sgRNA1 and sgRNA2) are shown. Nucleotide and amino acid sequences in red indicate the deleted region. The protospacer adjacent motifs (PAM) "NGG" are in blue. cDNA sequencing showed that genome editing did not cause a frameshift in the downstream sequence. (**c**), Representative

kymographs of pollen tubes generated from time-lapse imaging at 3 sec intervals for 450 sec. Pollen tube was excited by 514 nm Argon laser and emission wavelength at 517-589 nm was acquired. (**d**) Bright field images showing putative clear zones at *AtVPS13a∆C2:Venus* pollen tube tip. (**e**), Box plot of average distance of peak averaged Venus signal from pollen tube tip, see material and method for detail, the number below each box plot represent the number of pollen tube (*n*) analyzed; *p* value calculated from two-tailed Student's t-test; **, $p<0.01$. For box plots, center line shows the median, box limits indicate the 25th and 75th percentiles, whiskers extend 1.5 times the interquartile range from the 25th and 75th percentiles. (**f**), Percentage of ungerminated pollen after 45 min in an *in vivo* pollination assay; *n* indicates the number of pollination events. (**g**), Western blot analysis of crude protein extracted from mature pollen grains. Extracted proteins were roughly separated into supernatant/pellet fractions by 15,000xg 30 min centrifugation and anti-GFP primary antibody was used to detect tagged proteins.

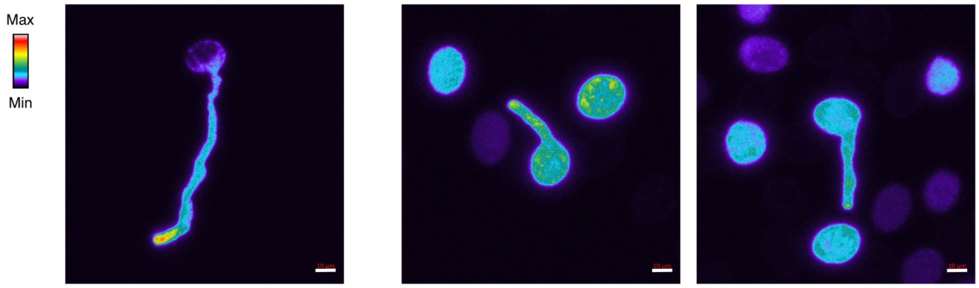

WT-AtVPS13a / Venus:RabA4B *atvps13a / Venus:RabA4B*

Fig. S12 Mis-localization of RabA4B vesicles in *atvps13a* pollen tubes. Images of RabA4B signal in *Arabidopsis thaliana* WT-*AtVPS13a* or *atvps13a* pollen tubes. The apical concentration of RabA4B is impaired in the mutant pollen tubes. Scale bars = 10 µm.

All supporting tables and movies are uploaded as separate files

Table S1 List of oligonucleotides used in this study.

Table S2 List of protein databases used to retrieve VPS13 sequence of each species.

Table S3 *List of detected proteins in selected sucrose fractions and their Pearson correlation coefficient with Arabidopsis thaliana VPS13a.*

Table S4 Result of PANTHER GO-Slim Biological Process analysis of proteins with high (>0.8) Pearson correlation coefficient with Arabidopsis thaliana VPS13a.

Table S5 List of proteins uniquely found in Arabidopsis thaliana VPS13a:Venus Co-IP sample and their Pearson correlation coefficients with AtVPS13a:Venus in sucrose gradient fractions proteomics.

Video S1 Live imaging of *in vitro* pollen germination of gene targeting AtVPS13a:Venus pollen

Video S2 Live imaging of *in vitro* pollen tube growth of AtVPS13a:Venus and endocytic vesicle marker FM4-64 dye

Video S3 Live imaging of *in vitro* pollen germination of dual reporter line gene targeting AtVPS13a:Venus and mCherry:RabA4B

Video S4 Live imaging of *in vitro* pollen germination of dual reporter line gene targeting AtVPS13a:Venus and Ca^{2+} sensor R-GECO1 showing AtVPS13a:Venus and Ca^{2+} relationship with cell wall deposition at the polarized site

Video S5 *In vivo* pollen Ca^{2+} spike during pollen hydration shown by ratiometric YFP/CFP video of pollen expressing YC3.6 calcium reporter

Video S6 Live imaging of *in vitro* pollen germination assay showing polarization of secretory vesicle marker RabA4B in *AtVPS13a* pollen grain and mis-localized RabA4B signal in *atvps13a* pollen grain

Supplemental Reference Lists

Gautier R, Douguet D, Antonny B, Drin G. **2008**. HELIQUEST: A web server to screen sequences with specific α-helical properties. *Bioinformatics* **24**: 2101–2102.

Kelly LA, Mezulis S, Yates CM, Wass MN, Sternberg MJE. **2015**. The Phyre2 web portal for protein modeling, prediction and analysis. *Nature Protocols* **10**: 845–858.

Klepikova AV, Kasianov AS, Gerasimov ES, Logacheva MD, Penin AA. 2016. A high resolution map of the Arabidopsis thaliana developmental transcriptome based on RNA-seq profiling. *Plant Journal* **88**: 1058–1070.

Pettersen EF, Goddard TD, Huang CC, Couch GS, Greenblatt DM, Meng EC, Ferrin TE. **2004**. UCSF Chimera - a visualization system for exploratory research and analysis. *Journal of Computational Chemistry* **25**: 1605–1612.
